# A “3+2” Cooperation Pattern of Amphipathic AIE Phototheranostic System for Multimodal Image‐Guided Synergistic Type I/II Photodynamic‐Photothermal Therapy

**DOI:** 10.1002/advs.202507956

**Published:** 2025-09-11

**Authors:** Haijun Ma, Yibo An, Yuanyuan Han, Feifan Zhao, Yunfei Zuo, Guokang He, Zhixiang Lu, Ryan T. K. Kwok, Jianwei Sun, Jacky W. Y. Lam, Yen Wei, Ben Zhong Tang

**Affiliations:** ^1^ Key Lab of Ministry of Education for Protection and Utilization of Special Biological Resources in Western China School of Life Sciences Ningxia University Yinchuan 750021 P. R. China; ^2^ State Key Laboratory of Cellular Stress Biology & Fujian Provincial Key Laboratory of Innovative Drug Target Research School of Pharmaceutical Sciences Xiamen University Xiamen 361102 P. R. China; ^3^ Department of Chemistry Hong Kong Branch of Chinese National Engineering Research Center for Tissue Restoration and Reconstruction Division of Life Science State Key Laboratory of Molecular Neuroscience and Department of Chemical and Biological Engineering The Hong Kong University of Science and Technology Clear Water Bay, Kowloon Hong Kong 999077 P. R. China; ^4^ School of Science and Engineering Shenzhen Institute of Aggregate Science and Technology The Chinese University of Hong Kong Shenzhen (CUHK‐Shenzhen) Guangdong 518172 P. R. China; ^5^ MOE Key Laboratory of Bioorganic Phosphorus Chemistry and Chemical Biology Department of Chemistry Tsinghua University Beijing 100084 P. R. China

**Keywords:** amphipathic aggregation‐induced emission, multimodal imaging, phototheranostic, photothermal therapy, Type I/II photodynamic therapy

## Abstract

Phototheranostic agents (PTA) control the process of phototheranostics for cancer. However, the existing PTA still cannot meet the needs of practical biomedical applications. Hence the development of new PTA will be imperative. In this contribution, a “3+2” cooperation pattern of amphipathicity PTA (named TDTMSB) with aggregation‐induced emission (AIE) features is designed and synthesized to perform near‐infrared I/II fluorescence imaging, photoacoustic imaging, photothermal imaging, and Type I/II photodynamic therapy (PDT)‐photothermal therapy (PTT) synergistic phototherapy for malignant tumors. Briefly, the target products (TMSB, TTMSB and TDTMSB) are successfully prepared by the reaction of 2‐methyl‐1‐(3‐sulfonatepropyl)‐benzothiazolium (MSB) and triphenylamine derivatives. They have high rotor twisted structure, doner‐acceptor (D‐A) conformation and unique AIE performances. Compared to TMSB and TTMSB, the TDTMSB exhibits a long near infrared emission that extends into the NIR II region, large Stokes shift (300 nm) and small energy gap. Simultaneously, it shows high ROSs generation capacity (^1^O_2_, OH, and O_2_^−^), photothermal conversion efficiency (up to 40.5%), and specific recognition of lysosomes in tumor cells. Furthermore, TDTMSB not only exhibits excellent multimodal imaging capabilities, but also carry out Type I/II PDT‐PTT synergistic enhancement effect for malignant tumor. Therefore, TDTMSB is expected to be a promising PTA for multimodal image‐guided phototherapy of tumors.

## Graphical abstract

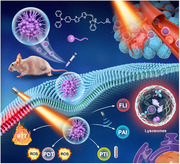

## 1 Introduction

The phototheranostic system, as an emerging key nanotechnology, plays an important role in real‐time bioimaging, diagnostic monitoring, and in situ treatment of serious hazards to human health major refractory diseases.^[^ ^1^ ^]^ Thereinto, bioimaging diagnostic methods mainly includes fluorescence imaging (FLI), photoacoustic imaging (PAI), and photothermal imaging (PTI).^[^ ^2^ ^]^ Fluorescence imaging has been widely used in biomedicine and clinical medicine, including subcellular localization analysis and fluorescence surgical navigation, which is mainly attributed to its numerous advantages, such as non‐invasive, high sensitivity, fast and efficient, low‐cost, real‐time monitoring, and multiplexing capability.^[^ ^3^ ^]^ However, it has some limitations containing relatively low spatial resolution and limited tissue penetration depth. Photoacoustic imaging (PAI), as another diagnostic approach, is able to supplement the deficiency of fluorescence imaging due to its excellent properties such as high spatial resolution, temporal resolution, and tissue penetration.^[^ ^4^ ^]^ It suffers only from low sensitivity, which can be complemented by fluorescence imaging. Therefore, the synchronous FLI‐PAI can be used as an effective way to enhance the diagnostic efficiency and realize the complementary advantages.^[^ ^5^ ^]^ Phototherapy, as a new type of non‐invasive tumor treatment, is mainly divided into two kinds of photodynamic therapy (PDT) and photothermal therapy (PTT).^[^ ^6^ ^]^ PDT is mainly produced by photosensitizer through energy conversion to produce singlet oxygen (^1^O_2_) or by electron transfer to form free radical species (·OH, O_2_^─^, and H_2_O_2_ etc.) damage biological macromolecules, which are respectively called Type I PDT and Type II PDT, even the Type II PDT requires not only photosensitizer and energy source, but also adequate oxygen.^[^ ^7^ ^]^ However, the microenvironment of malignant tumors is an unusual hypoxic characteristic, which limits PDT effect on cancer. Type I PDT has become the first choice for PDT because it is not restricted by oxygen.^[^ ^8^ ^]^ Also, PTT, as another momentous phototherapy, can effectively accelerate the blood circulation of the irradiated tumor site, which can further improve the problem of tumor hypoxia and oxygen consumption in PDT.^[^ ^9^ ^]^ Therefore, the PDT‐PTT technology formed by the combination of PDT and PTT can greatly improve the phototherapy effect.^[^ ^10^ ^]^ Phototheranostic agents are the most critical elements to achieve high‐efficiency phototherapy effects.^[^ ^11^ ^]^ Until now, some phototheranostic agents have been designed and applied to the biological system, such as chlorin e6 (Ce6), boron dipyrromethene (BODIPY) and porphyrins derivatives, indocyanine green (ICG), and quantum dots, etc.^[^ ^12^ ^]^ These phototheranostic agents exhibited better therapeutic effects against tumors in the single molecule state. However, there are many limitations to their application in the aggregated state, including aggregation‐caused quenching (ACQ) effects duo to intermolecular π−π interactions, low sensitivity and ROS yield, and unsatisfactory therapeutic effect. Therefore, a novel phototheranostic agent with high‐performance there is an urgent need for anti‐tumor applications.

Aggregation‐induced emission (AIE) is an anti‐ACQ photophysical phenomenon in which organic fluorescent molecules achieve fluorescence enhancement in aggregated or solid states. Since Tang et al. proposed the concept of AIE in 2001, a large number of AIE molecules have been studied.^[^ ^13^ ^]^ Such fluorescent materials display an extremely weak fluorescent signal in dilute solution, but they exhibited a bright fluorescence signal in aggregate state or solid state. Also, AIE materials possess excellent characteristics including large stokes shift, against photobleaching capability, high photostability, and ROSs generation yield, which led to its wide application in the biomedical field.^[^ ^14^ ^]^ In particular, AIE molecules, as theranostic agents, have been widely concerned and developed for the real‐time diagnosis, monitoring, and treatment of malignant tumors. However, the development of multipurpose AIE phototheranostic systems with high performance are still rare, and there are immense challenges.^[^ ^15^ ^]^ This is mainly because the multifunctional AIE system with excellent performance should be able to achieve FLI, PAI, PTT, and PDT simultaneously.^[^ ^16^ ^]^ In fact, there is a competition between their radiative and nonradiative decay processes according to the Jablonski diagram.^[^ ^17^ ^]^ Their balance can be tuned by introducing rotors into organic molecules, which is also one of the pivotal elements in the design and development of phototheranostic agents. AIE fluorophores with D‐π‐A conformation are expected to fit the bill, which mainly due to the following reasons. First, AIE fluorophore with D‐π‐A conformation have unique photometric characteristics including anti‐ACQ ability, high absorption and emission wavelengths, large Stokes shift, etc. These excellent properties are more conducive to high‐resolution fluorescence imaging and photoacoustic imaging.^[^ ^18^ ^]^ Second, it is capable of promoting the HOMO and LUMO orbital separation and boosting the intersystem crossing (ISC) process, which effectively improves the production of ROSs.^[^ ^19^ ^]^ Third, twisted conformations of AIE fluorophores with D‐π‐A structures can not only attenuate intermolecular π−π stacking interactions but also increase the relaxation degree of molecular. Part of the molecular rotors are still free to rotate even in the aggregated state, so as to effectively regulate the non‐radiative decay.^[^ ^20^ ^]^ Therefore, balancing the energy consumption of radiative and non‐radiative transitions can greatly improve the comprehensive efficacy of phototheranostic agents, and is also a promising strategy for the development of multipurpose phototheranostic systems.^[^ ^21^ ^]^

**Scheme 1:**
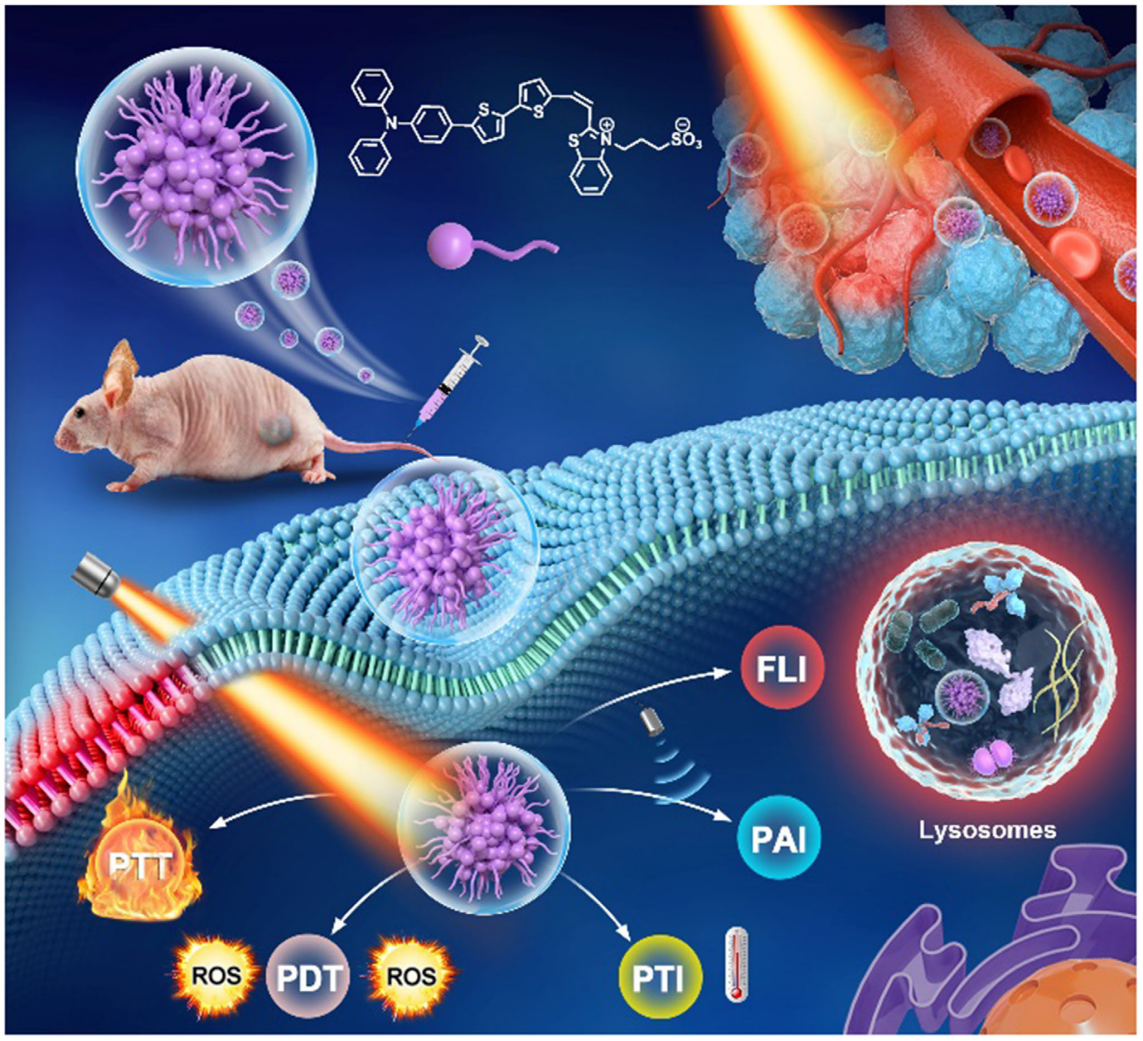
Schematic illustration of TDTMSB NPs for image‐guided Type I/II PDT‐PTT synergistic phototherapy.

In this work, we present a novel multipurpose amphiprotic PTA with AIE characteristics to carry out the “3+2” cooperation pattern of synergistic phototheranostic for cancer (**Scheme** **1**). Briefly, propanesultone and 2‐methylbenzothiazole form 2‐methyl‐1‐(3‐sulfonatepropyl)‐benzothiazolium (MSB) via a ring‐opening reaction. Subsequently, it reacts with 4‐(N,N‐diphenylamino)benzaldehyde, 5‐(4‐(diphenylamino)phenyl)thiophene‐2‐carbaldehyde, and 5'‐(4‐(diphenylamino)phenyl)‐[2,2'‐bithiophene]‐5‐carbaldehyde to prepare target products (TMSB, TTMSB, and TDTMSB) through a classical Knovenagel reaction. The as‐prepared TDTMSB has a high rotor twisted structure and D‐A conformation, which promote its AIE performances, long NIR emission, and large Stokes shift (up to 300 nm). Simultaneously, TDTMSB is dissolved in a DMSO/H_2_O mixture solution to form NPs through a simple self‐assembly process and is named TDTMSB NPs. Such NPs show good biocompatibility, high stability, meritorious ROSs production capacity (^1^O_2_, OH, and O_2_^─^) and photothermal conversion efficiency (40.5%). In vitro demonstrated that TDTMSB enables perform good fluorescence imaging and specific targeting the lysosome in tumor cells. Also, it can effectively inhibit the growth of tumor cells and regulate the expression of apoptosis related factors. Furthermore, in vivo experiment results displayed that TDTMSB not only can realize highly sensitive NIR I/II fluorescence imaging, high‐resolution photoacoustic imaging, and photothermal imaging, but also perform excellent image‐guided Type I/II PDT‐PTT synergistic phototherapy for malignant tumors. Therefore, TDTMSB can be used as a potential candidate for multimodal phototheranostic of malignant tumors, and it provides a new design strategy for the development of versatile phototheranostic agents.

## 2 Results and Discussion

### 2.1 Design, Synthesis, and Characterization of Phototheranostic Materials

**Figure 1:**
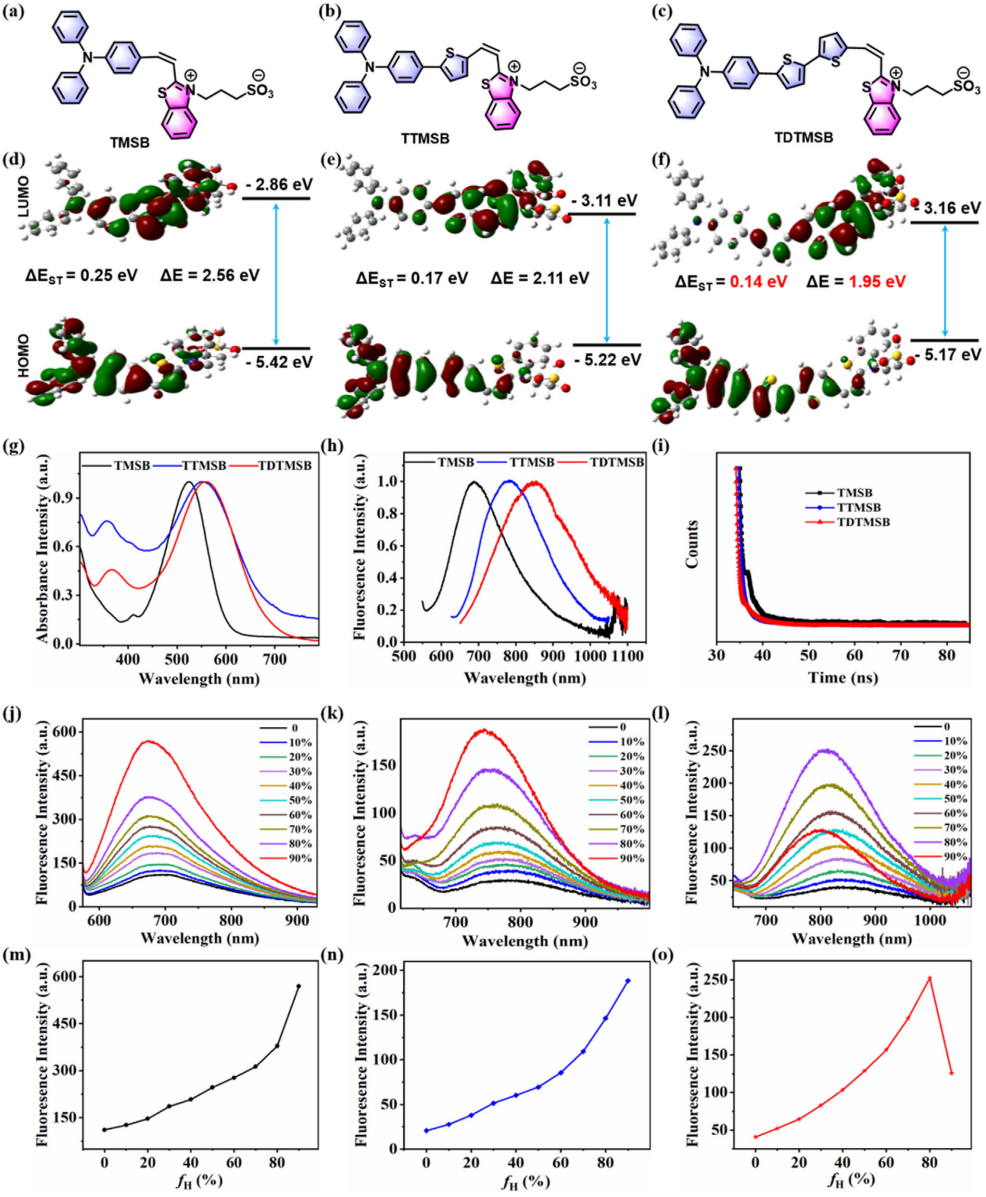
Chemical structures of a) TMSB, b) TTMSB, and c) TDTMSB compound. The HOMO‐LUMO orbital energy gaps and the energy level values from the singlet to triplet states of d) TMSB, e) TTMSB, and f) TDTMSB are calculated using time‐dependent density functional theory (TD‐DFT) at the level of B3LYP/6‐31G^*^. g) Absorption spectrum, h) Fluorescent emission spectrum, i) Fluorescence lifetime, j–l) Fluorescent emission intensity in different proportions of ethanol/n‐hexane mixture, and m–o) AIE characteristics curve the target products (TMSB, TTMSB, and TDTMSB).

The development of high performance phototheranostic agent is crucial to the efficacy of the entire phototheranostic process. An optimal phototheranostic agent should exhibit a combination of key advantages, including strong absorption, long emission wavelength, large Stokes shift, high ROSs generation capacity, and photothermal conversion efficiency.^[^ ^22^ ^]^ However, the integration of strong electron donor and acceptor groups, coupled with extended π‐conjugate bridges, significantly enhances the absorption and emission properties of the fluorescent molecules. Simultaneously, some phototheranostic agents with D‐A structures have demonstrated enhanced ROSs generations and improved photothermal conversion efficiency.^[^ ^23^ ^]^ Triphenylamine (TPA) is incorporated into this system for its potent electron‐donating properties, which effectively redshifts t the molecule emission wavelength. And its non‐planar spatial structure mitigates ACQ effect by suppressing *π–π* interaction. Thiophene, as an electron *π*‐conjugate bridge, can enhance orbital delocalization between donor and acceptor, thereby reducing the energy gap of the highest occupied molecular orbital (HOMO) and lower unoccupied molecular orbital (LUMO) and diminishing singlet‐triplet splitting energy (ΔΕ_ST_) values. Notably, extending *π*‐bridge can be achieved by increasing the number of thiophene units, which prompts the molecular emission wavelength redshift, reduces ΔΕ_ST_ value, and boosts ROSs generation. 2‐methylbenzothiazole is selected as the electron acceptor due to its strong electron‐accepting capabilities and potential for further functional modifications, which are mainly attributed to the high reactivity of the nitrogen atom in the benzothiazole. Furthermore, sulfonate and quaternary ammonium salt with amphipathicity are introduced into this system, significantly enhancing biocompatibility, reducing toxicity, and extending the long emission wavelength of fluorescence materials.^[^ ^24^ ^]^ In accordance with the above design strategies, we present the development and systematic investigation of a high‐performance multimodal phototheranostic agent designed for accurate cancer theranostics. And the synthesis procedure is illustrated in Figure S1 (Supporting Information). Specifically, the precursor is prapared from propanesultone and 2‐methylbenzothiazole through a brief ring‐opening reaction and named 2‐methyl‐1‐(3‐sulfonatepropyl)‐benzothiazolium (MSB). It then undergoes a Knoevenagel coupling reaction with triphenylamine‐based derivatives ((4‐(N,N‐diphenylamino)benzaldehyde, 5‐(4‐(diphenylamino)phenyl)thiophene‐2‐carbaldehyde, and 5'‐(4‐(diphenylamino)phenyl)‐[2,2'‐bithiophene]‐5‐carbaldehyde)) to form the propeller‐shaped target products (TMSB, TTMSB, TDTMSB) with stable double bonds (**Figure** **1**a,c). Immediately, we utilized NMR spectroscopy and MALDI‐TOF‐MS to verify the chemical structure and determine the molecular weight of target compounds (Figures S2–S8, Supporting Information). To further evaluate the performance of the synthesized target products, time‐dependent density functional theory (TD‐DFT) calculations were employed to optimize and estimate their molecular conformations. The calculated results are shown in Figure 1d,f, the HOMO‐LUMO gaps of target products (TMSB, TTMSB, TDTMSB) are ≈−2.86, −3.11, −3.16, −5.42, −5.22, and −5.27 eV, respectively. Their energy gap (ΔΕ) are ≈2.56, 2.11, and 1.95 eV, with a small singlet‐triplet splitting energy (ΔΕ_ST_) of 0.25, 0.17, and 0.14 eV. Remarkably, the calculated outcomes align with our design strategy, indicating that TDTMSB is readily activated and has generates high ROSs generation capacipy. This primarily attributes to its smallest ΔΕ (1.95 eV) and ΔΕ_ST_ (0.14 eV) values compared to TMSB and TTMSB.

Next, the optical properties of the synthesized target products (TMSB, TTMSB, and TDTMSB) are investigated using a UV−vis spectrophotometer and a Duetta fluorescence spectrophotometer. As shown in Figure 1g,i, their optimum absorption and emission wavelengths in ethanol are ≈525, 550, 560, 690, 770, and 860 nm, respectively. The Stokes shifts of these fluorescent molecules are ≈165, 220, and 300 nm. Their fluorescence lifetimes in ethanol are ≈302, 732, and 548 ps. Notably, when these target compounds are dissolved in ethanol/n‐hexane or in the aggregate state, their fluorescence lifetime significantly increase (TMSB for 816 ps, TTMSB for 1067 ps, and TDTMSB for 1976 ps) in Figure S9 (Supporting Information). Subsequently, AIE properties of the synthesized products are verified through an investigation of their emission spectra in various ethanol/n‐hexane ratios. As depicted in Figure 1j,o, a progressive enhancement in fluorescence intensity was observed as the n‐hexane fraction increased, this phenomenon primarily due to the augmented aggregation propensity of the target molecules facilitated by the higher n‐hexane concentration. The restricted rotation of the benzene ring on the donor and benzothiazole on the acceptor suggests that the target products (TMSB, TTMSB, and TDTMSB) possess unique AIE features. Noteworthily, for the TDTMSB molecule, increasing the n‐hexane/ethanol ratio from 0% to 80% resulted in a gradual enhancement of fluorescence intensity. However, when the ratio reached 90%, the fluorescence emission intensity decreased significantly. It is primarily attributed to the larger rigid structure of TDTMSB, which promotes enhanced aggregation and even leads to the precipitation of some aggregates. Based on the above analysis, TDTMSB exhibits the smallest ΔΕ (1.95 eV) and ΔΕ_ST_ (0.14 eV) values, along with superior optical properties compared to TMSB and TTMSB. Therefore, the TDTMSB molecule is preferred for subsequent experiments.

### 2.2 Synthesis and Characterization of TDTMSB NPs

**Figure 2:**
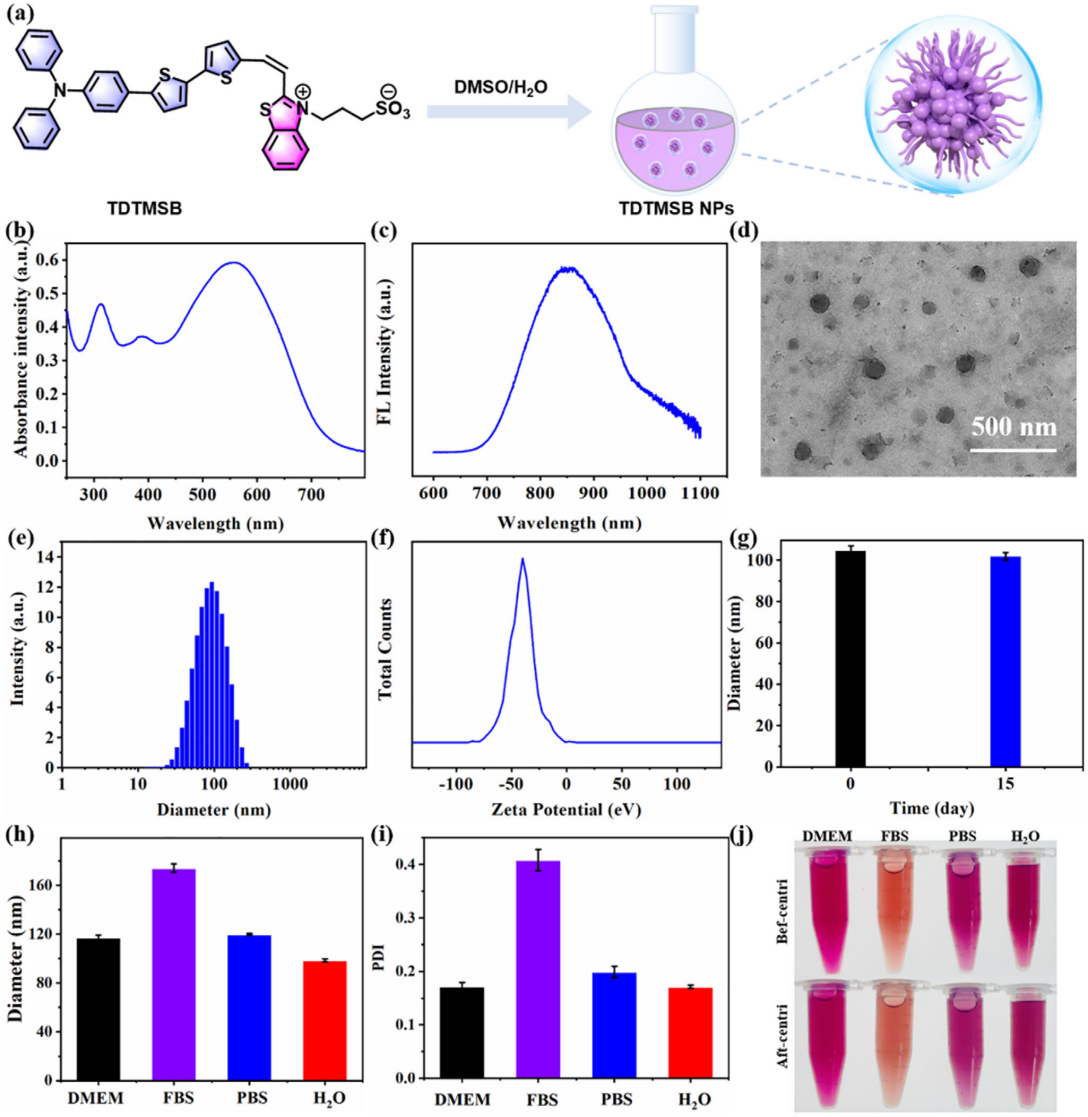
a) Preparation process of TDTMSB NPs b) Absorption spectrum, c) emission spectrum, d) transmission electron microscopy image, e) hydrodynamic diameter distribution, and f) Zeta potential of TDTMSB NPs. The scale bar is 500 nm. g) Average hydrodynamic diameter of TDTMSB NPs at 4 °C conditions for 0 days and 15 days. h) The hydrodynamic diameter, i) polydispersity index (PDI) of TDTMSB NPs in various media (DMEM, FBS, PBS, and H_2_O), j) optical photograph of TDTMSB NPs in different medium (DMEM, FBS, PBS, and water) before and after centrifugation at 5000 rpm for 10 min.

To facilitate the application of TDTMSB in biological systems, enhancing its biocompatibility and stability is imperative. TDTMSB was thus formulated into nanoparticles (TDTMSB NPs) via a straightforward self‐assembly process, involving dissolution in a DMSO/H_2_O blend followed by ultrasonication (**Figure** **2**a). Subsequently, the optical properties of these TDTMSB NPs were meticulously examined utilizing UV–vis spectroscopy and fluorescence spectroscopy techniques. As shown in Figure 2b,c and Figure S10 (Supporting Information), the TDTMSB NPs exhibit an absorption peak at 560 nm and a fluorescence emission maximum at 865 nm, resulting in a substantial Stokes shift of 305 nm. And fluorescence lifetime is ≈421 ps. TEM images indicates that TDTMSB NPs possess a narrow size distribution (40–50 nm). DLS analysis further indicates a reduced hydrodynamic diameter distribution (101.83 ± 1.88 nm), a low polydispersity index (0.175), and a negative zeta potential (−40.74 mV). These characteristics suggest that the TDTMSB NPs are optimally sized for cellular uptake and exhibit good stability (Figure 2d,f). Subsequently, the stability of TDTMSB NPs is further verified. The hydrodynamic diameter distribution of TDTMSB NPs in various media (H_2_O, PBS, FBS, and DMEM) remained ≈100 nm with a low polydispersity index. Additionally, the NPs maintained a uniform transparent solution, with no precipitation observed before or after centrifugation. When stored at 4 °C for 15 days, the hydrodynamic diameter of TDTMSB NPs showed no significant changes (Figure 2g,j). Moreover, the TDTMSB NPs are irradiated with a continuous 560 nm laser to assess their stability. The relative fluorescence signals remained above 90% (Figure S11, Supporting Information), indicating that the TDTMSB NPs exhibit excellent biocompatibility and stability, highlighting their significant potential for biological.

### 2.3 ROSs Generation Capability and Photothermal Effects of TDTMSB NPs

**Figure 3:**
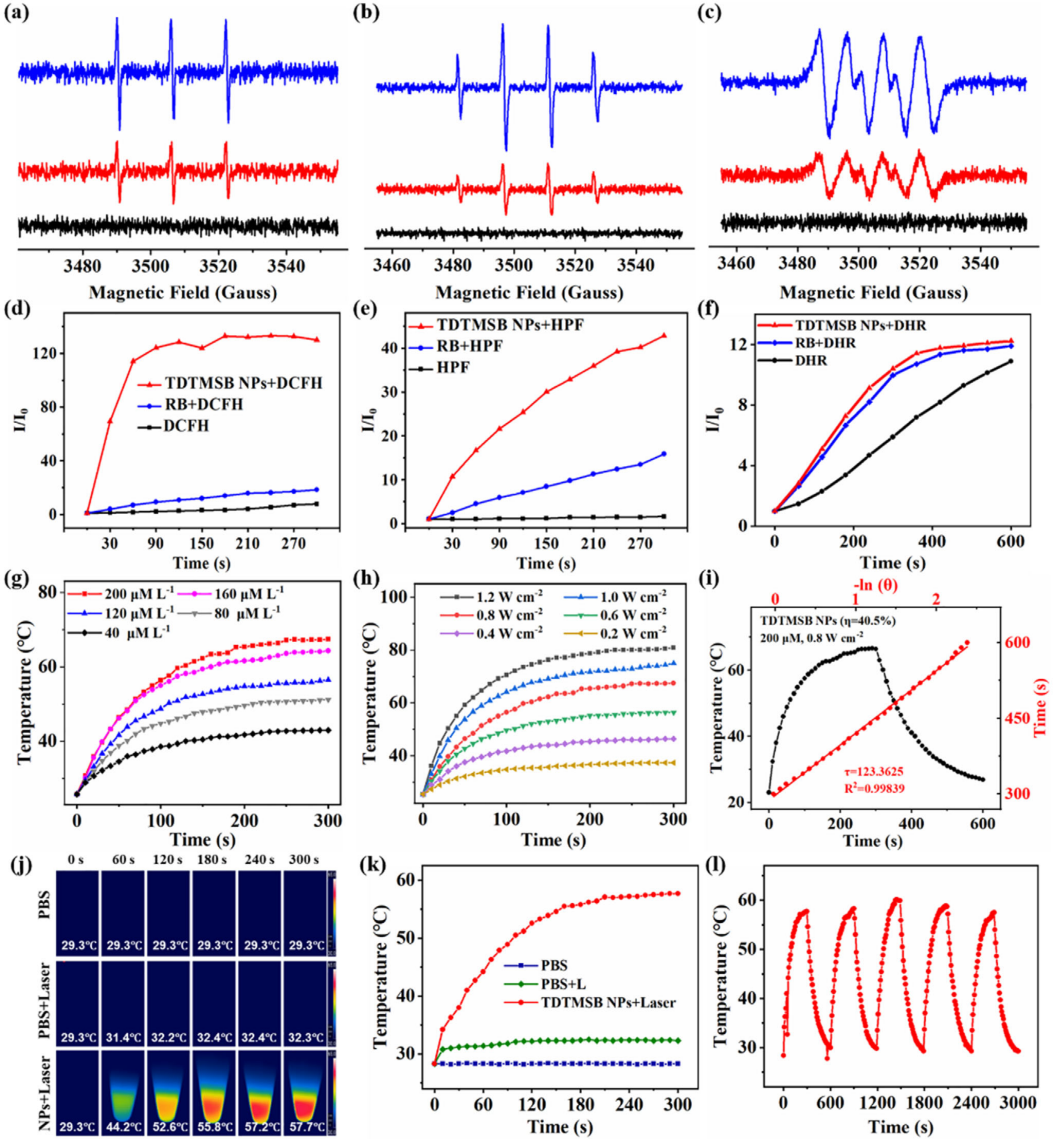
The ROS generation capability and photothermal conversion efficiency of TDTMSB NPs. Electron spin resonance (ESR) spectra signals of a) singlet oxygen (^1^O_2_) b) hydroxyl radical (·OH), and c) superoxide radical (·O_2_^−^) generated by TDTMSB NPs under dark/light conditions (the black curve represents the absence of light, the red curve and blue curve represent the sample being illuminated by light for 2 and 4 min, respectively). d) The total ROSs generation ability of TDTMSB NPs is determined by the DCFH‐DA probe. The hydroxyl radical (·OH) e) and superoxide radical (·O_2_^−^) f) production capability of such NPs is detected using the HPF assay and DHR fluorescence probe. g) the temperature change of TDTMSB NPs with different concentrations are irradiated by a 635 nm laser (0.8 W cm^−2^) h) the temperature change of TDTMSB NPs is illuminated through a laser with various intensity. i) Photothermal conversion efficiency of TDTMSB NPs j) The photothermal images and k) photothermal curve of PBS, PBS+laser, and TDTMSB NPs+Laser. l) The photothermal cycle curve of TDTMSB NPs.

Next, we assessed the ROS production capability of TDTMSB NPs and utilized electron paramagnetic resonance (EPR) spectroscopy to determine the specific ROS species.^[^ ^25^ ^]^ As illustrated in **Figure** **3**a,c, TDTMSB NPs exhibit no EPR signals under dark conditions. However, when exposed to white light for 4 min in the presence of 2,2,6,6,‐tetramethylpiperidine (TEMP), EPR analysis revealed three distinct equimolar peaks characteristic of TEMPO formation. The signal intensity increased progressively with illumination duration. Then, the EPR signal of TDTMSB NPs mixed with 5,5‐dimethyl‐1‐pyrroline N‐oxide (DMPO) is detected under light irradiation. The results show four peaks with an intensity ratio of 1:2:2:1. However, when TDTMSB NPs were mixed with 5‐tert‐butoxycarbonyl‐5‐methyl‐1‐pyrroline N‐oxide (BMPO) and illuminated, six peaks were observed. The peak intensities at positions 1, 2, 4, and 6 were approximately equal, while the peaks at positions 3 and 5 showed slightly reduced intensity. EPR results indicated that the TDTMSB NPs are capable of producing substantial amounts of ROSs, including singlet oxygen(^1^O_2_), hydroxyl radical (·OH), and superoxide radical (·O_2_^−^). Furthermore, the total ROSs production efficiency of TDTMSB NPs is determined by a DCFH probe (Figure 3d). The results demonstrated that the DCFH group exposed to light for 300 s exhibits no significant change, whereas the relative fluorescence intensity (I/I_0_) of the RB+DCFH group progressively increased with prolonged illumination time. Upon irradiation for 300 s, the I/I_0_ values reached as high as 18, suggesting that RB can generate ROSs. Notably, the I/I_0_ values of TDTMSB NPs + DCFH group dramatically increased to 130 upon illumination for just 90 s. And as the irradiation time was extended from 90 to 300 s, the I/I_0_ value remains constant, showing no conspicuous change. These results demonstrat that TDTMSB NPs possess a higher yield of ROSs generation compared to the RB agent. Simultaneously, we selected Rose Bengal (RB) work as a standard agent and quantitatively calculated the singlet oxygen (^1^O_2_) production efficiency of TDTMSB NPs by measuring the decay rate of 9,10‐Anthracenediyl‐bis(methylene)‐dimalonic acid (ABDA). As shown in Figure S13 (Supporting Information), the ^1^O_2_ generation efficiency of TDTMSB NPs exceeds 80%, surpassing that of RB at 78%. Additionally, the production yield of the hydroxyl radical (·OH) and superoxide radical (·O_2_^−^) are recorded using the hydroxyphenyl fluorescein (HPF) and dihydrorhodamine123 (DHR123) fluorescence probes (Figure 3e,f). Experimental results indicate that TDTMSB NPs generate a significantly higher amount of OH and O_2_^−^ compared to RB. Moreover, the dichloro‐dihydro‐fluorescein diacetate (DCFH‐DA) assay was performed to determine whether the TDTMSB NPs could induce ROSs formation in vitro (Figure S12, Supporting Information). Experimental findings revealed that tumor cells treated with TDTMSB NPs are labeled and displayed a bright green fluorescent signal under the light irradiation, and no green fluorescent signals are observed in the control group. This demonstrates that TDTMSB NPs effectively generate significant quantities of ROS, including ^1^O_2_, OH, and O_2_^−^, both within and outside the cell. Besides, the photothermal conversion efficiency of TDTMSB NPs are further investigated by an infrared thermal imaging system. Concretely, temperature variations in TDTMSB NP at different concentrations were monitored under varying irradiation intensities from a 635 nm laser. As showcased in Figure 3g,h, a marked increase in temperature is observed for TDTMSB NPs with both increasing concentration and higher illumination power densities. Subsequently, the temperature change of the sample was comprehensively investigated at a concentration of 120 µm and a light intensity of 0.8 W cm^−2^. As displayed in Figure 3i,l, the initial temperatures of both TDTMSB NPs and PBS were measured at 29.3 °C under room temperature conditions. Upon irradiation with a 630 nm laser (0.8 W cm^−2^) for 0–300 s, the temperature of PBS rose from 29.3 to 32.4 °C within 180 s, which it exhibited no significant change for the remaining 180–300 s. Overall, the temperature increase in PBS under laser exposure was only 3–5 °C compared to its initial state without irradiation. Interestingly, the temperature of TDTMSB NPs dramatically increased from 29.3 to 55.8 °C when exposed to a 630 nm laser for 180 s. Furthermore, continuing the laser exposure for an additional 120 s raised the temperature to 57.7 °C. Additionally, TDTMSB NPs are able to undergoing multiple photothermal cycles without a significant decrease in temperature during subsequent cycles compared to the first cycle. Moreover, the photothermal conversion efficiency (PCE) of TDTMSB nanoparticles in ultrapure water was calculated and analyzed through temperature monitoring, revealing a maximum PCE of 40.5%. These data convincingly demonstrate that TDTMSB NPs not only exhibit a strong capacity for generating ROSs (^1^O_2_, OH, and O_2_^−^) but also possess excellent properties and thermal stability. Consequently, it is anticipated to serve as a highly efficacious phototheranostic agents in biomedicine.

### 2.4 Colocalization and Phototherapy Effect Evaluation of TDTMSB NPs In Vitro

**Figure 4:**
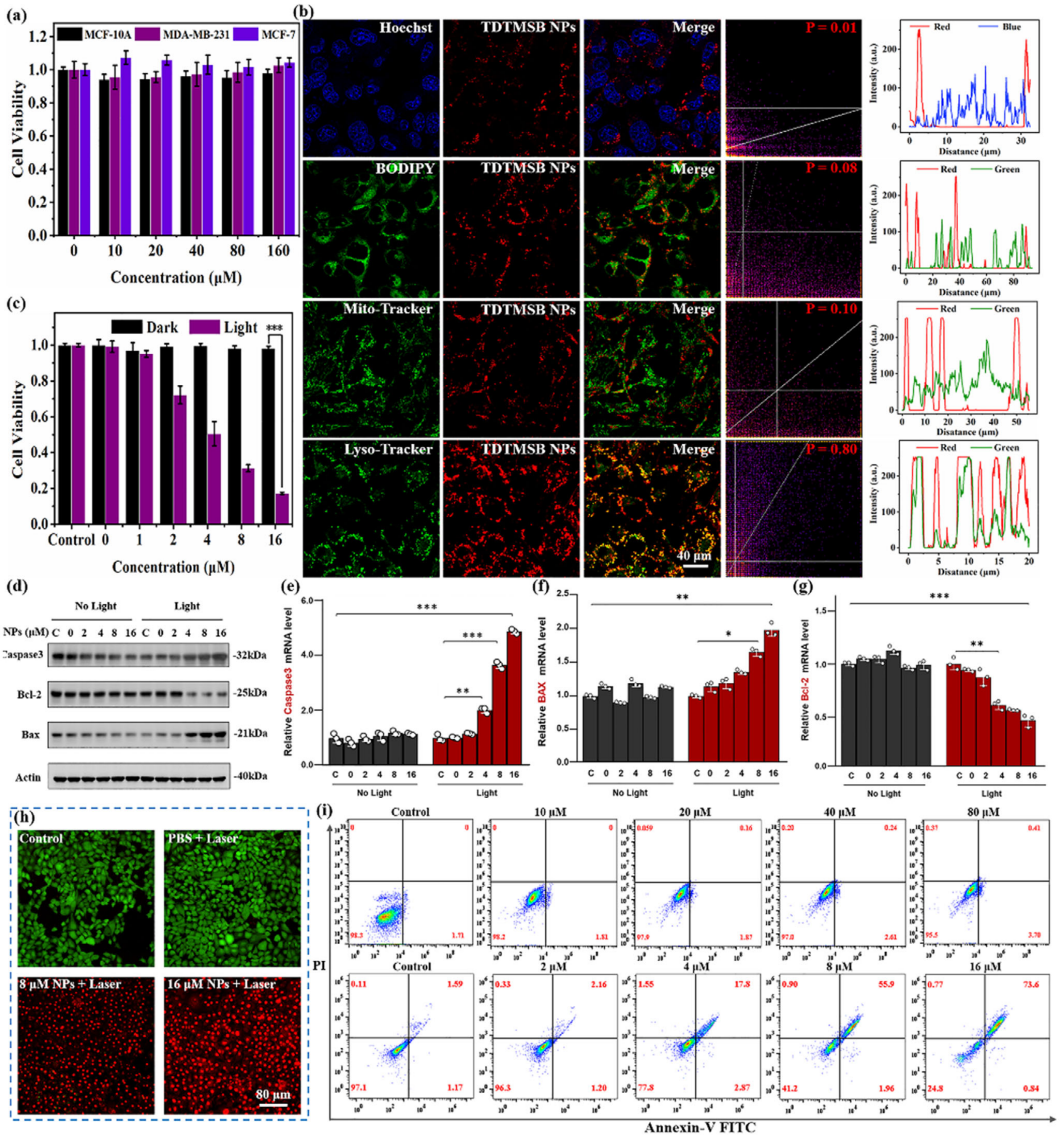
a) The cytotoxicity test of TDTMSB NPs is executed by using MCF‐10A, MDA‐MB‐231, and MCF‐7 cells. b) The colocalization analysis of subcellular organelles and Pearson correlation coefficient was performed by an CLSM imaging system (hoechst33258 for the nucleus, mito‐tracker green for the mitochondria, BODIPY493/503 for the liquid droplet, and lyso‐tracker green for the lysosomes). Scale bar = 40 µm. c) Cell viability of MDA‐MB‐231 cells is incubated with TDTMSB NPs at various concentrations under light conditions for 10 min, and collected by CCK‐8 kit. d) The expression of related apoptosis factors (Caspase3, Bax, and Bcl‐2) under different conditions is recorded by a Western blotting test. The expression of pro‐apoptosis factors Caspase3 e) and Bax f), and inhibitor of apoptosis factors Bcl‐2 g) in dark/light conditions are collected. ^*^ *p* < 0.05, ^**^ *p* < 0.01, and ^***^ *p* < 0.001. h) Live/dead dying assay of MDA‐MB‐231 cells is performed using a calcein‐AM/PI kit. The scale bar is 80 µm. i) The survival rate and apoptosis rate of MDA‐MB‐231 cells treated with TDTMSB NPs under both dark and light conditions through a flow cytometry assay.

Cytotoxicity, or the absence thereof, serves as a critical criterion in assessing the suitability of nanomaterials for biomedical applications.^[^ ^26^ ^]^ The CCK‐8 assay demonstrated that TDTMSB NPs exhibit no cytotoxicity at a concentration of 80 µm. Cell viability remained as high as 90%, when the cells (MCF‐10A, MCF‐7, and MDA‐MB‐231) were incubated with varying concentrations (0, 5, 10, 20, 40, and 80 µm) in the dark for 24 h (**Figure** **4**a). Also, the flow cytometry assay yielded consistent results with the CCK‐8 assay. Notably, the survival rate of MDA‐MB‐231 cells exceeded 90% when exposed to the NPs in the dark for 24 and 36 h, with an apoptosis rate of merely 7.82% (Figure S14, Supporting Information). This suggests that the TDTMSB NPs have negligible cytotoxicity at concentration below 80 µm. Thereby affirming their suitability for biosystem applications. Next, the in vitro fluorescent imaging of TDTMSB NPs was investigated using a confocal laser scanning microscopy (CLSM) system. To elucidate their intracellular localization post‐uptake, a colocalization analysis was conducted to ascertain the specificity of TDTMSB NPs toward subcellular compartments, employing CLSM. The experimental findings revealed minimal overlap in fluorescence signals between TDTMSB NPs and markers for nuclei (Hoechst 33258), mitochondria (Mito‐Tracker Green), and lipid droplets (BODIPY 493/503), with Pearson's correlation coefficients recorded at 1.0%, 8.0%, and 10.0%, respectively. These data suggest that TDTMSB NPs do not exhibit specific affinity for the cell nucleus, mitochondria, or lipid droplets. Interestingly, the fluorescent signals of both Lyso‐Tracker Green and TDTMSB NPs overlap almost completely, with a Pearson's correlation coefficient of up to 80.0% (Figure 4b). These findings indicate that TDTMSB accumulates in lysosomes upon entering cells and achieves precise recognition of lysosomes.

Given the exceptional cellular‐level fluorescent imaging capabilities of TDTMSB NPs, their phototherapeutic efficacy was thoroughly investigated. First, MDA‐MB‐231 cells incubated with varying concentrations of TDTMSB NPs underwent CCK‐8 assays under both dark and illuminated conditions to assess viability. As displayed in Figure 4c, the cell activity in the dark exhibited no distinct difference compared to the control group. However, under illumination, the cell viability significantly decreased with increasing concentrations of TDTMSB NPs. At a concentration of 16 µm, the tumor inhibition efficiency exceeded 80%. Second, flow cytometry experiments revealed a significant decrease in the survival rate of MDA‐MB‐231 cells treated with TDTMSB NPs under light conditions, and the apoptosis rate increased dramatically to 73.6% compared to the control group (Figure 4i). To further investigate the phototherapy efficacy of TDTMSB NPs, a live/dead dying assay was implemented. The strong green fluorescent signals were clearly observed in the control group, as the cells were stained with Calcein AM, indicating superior cell viability. Under light conditions, the cells incubated with TDTMSB NPs exhibited bright red fluorescence upon staining with propidium iodide. This effect primarily results from the photoactivation of TDTMSB NPs, leading to reactive oxygen species (ROS) generation and a significant amount of heat release, ultimately causing MDA‐MB‐231 cells damage (Figure 4h). Moreover, to explore the phototherapy effect of TDTMSB, the expression of related apoptosis factors (Caspase3, Bax, and Bcl‐2) was examined. As illustrated in Figure 4d,g, incubation of tumor cells with TDTMSB NPs in the absence of light did not yield significant alterations in these apoptotic factors. Interestingly, the treatment of tumor cells with such NPs under light conditions resulted in a dose‐dependent upregulation of pro‐apoptotic proteins, specifically caspase‐3 and Bax. Conversely, the expression of the inhibited apoptosis protein bcl‐2 significantly decreased with increasing NPs concentrations. These results indicate that TDTMSB NPs are able to modulate the expression of related apoptosis factors under light activation, highlighting their potent phototherapeutic efficacy.

### 2.5 In Vivo Multimodal Imaging and PDT‐PTT Synergistic Therapy of TDTMSB NPs

**Figure 5:**
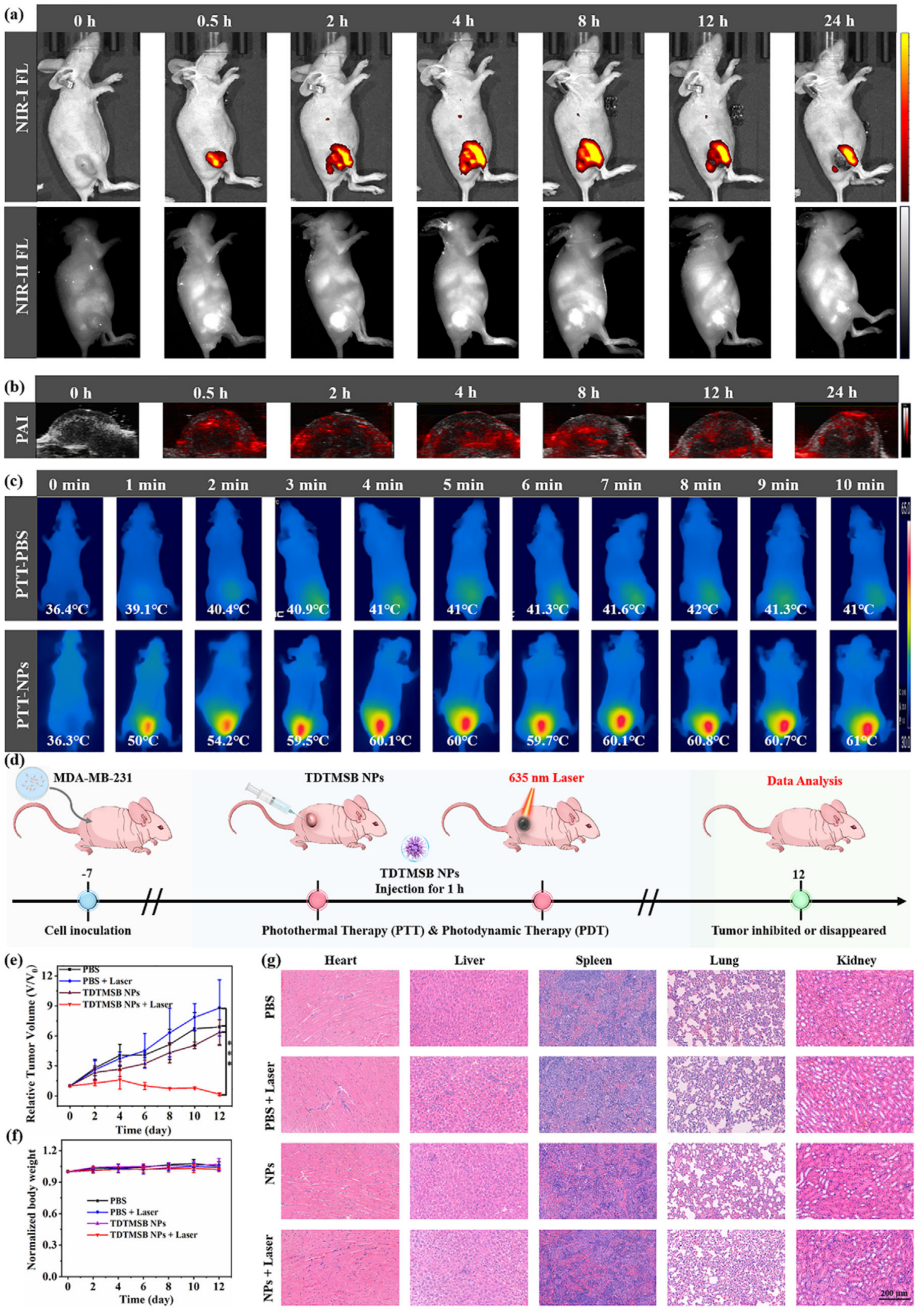
a) Near‐infrared I/II fluorescence imaging, b) photoacoustic imaging, and c) photothermal imaging of MDA‐MB‐231 tumor‐bearing BALB/c nude mice at different times after intratumor injection of TDTMSB NPs. d) The diagram of the experimental process in vivo. e) Tumor growth volume, f) bodyweight changes of tumor‐bearing mice (n = 6, mean ± S.D.), and g) H&E‐stained images of major organs (heart, liver, spleen, lung, and kidney) from tumor‐bearing mice treated with PBS, PBS + laser, TDTMSB NPs, and TDTMSB NPs + laser. The scale bar: 200 µm. ^*^ *p* < 0.05, ^**^ *p* < 0.01, and ^***^ *p* < 0.001 represent statistically significant difference.

To further investigate the excellent properties of TDTMSB NPs, in vivo bioimaging assays were performed. Fluorescent imaging results revealed that the bright red fluorescent signal in solid tumors of BALB/c nude mice was clearly observed by the IVIS system following intratumor injection at various time points (0.5, 1, 2, 4, 8, 12, and 24 h). Importantly, the strongest fluorescence intensity signal was recorded at 0.5 h post‐injection. As the injection time increased, the red fluorescent signal area continued to expand but weakened in intensity. By 24 h post‐injection, the red fluorescent signal had decreased to its weakest level (**Figure** **5**a). Subsequently, photoacoustic imaging was performed to illustrate the depth penetration capabilities of solid tumors (Figure 5b). The findings revealed that TDTMSB NPs facilitate high‐resolution photoacoustic imaging, exhibiting consistent trends and characteristics with fluorescence imaging results. Therefore, TDTMSB NPs demonstrate dual functionality: exceptional sensitivity in fluorescence imaging and superior depth resolution in photoacoustic imaging for solid tumor detection. Moreover, the photothermal imaging was conducted on MDA‐MB‐231 BALB/c nude mice after intratumor injection of TDTMSB NPs using a 635 nm laser irradiation for 10 min. Temperature changes in the irradiated region were determined at intervals utilizing an infrared thermal camera. The imaging results indicated a significant increase in tumor temperature from 35.7 to 60.1 °C upon laser exposure. In contrast, PBS‐treated mice exhibited no significant temperature change under identical conditions, reaching a maximum of 41.6 °C (Figure 5c; Figure S15, Supporting Information). This demonstrates that TDTMSB NPs possess strong photothermal performance and potential as a promising phototheranostic agent for tumor photothermal ablation.

Next, the PDT‐PTT treatment effect of TDTMSB NPs in vivo is evaluated using MDA‐MB‐231 tumor‐bearing BALB/c nude mice models. The mice were treated with PBS and TDTMSB NPs (100 µL) for 1 h, followed by illuminated with a 635 nm laser (300 mW cm^−2^) for 10 min. This treatment is repeated over seven cycles. The tumor volume in tumor‐bearing mice treated with the PBS, PBS + laser, and TDTMSB NPs gradually increases, indicating no inhibitory effect on tumor growth. However, the MDA‐MB‐231 tumor‐bearing mice conducted with TDTMSB NPs and laser showed significant improvements. The tumor volume gradually decreased, with some tumors completely crusted over or disappeared. Furthermore, after 15 days of treatment, solid tumors were collected from all the tumor‐bearing mice, and their sizes were recorded. The gross tumor volume was significantly reduced compared to other groups, suggesting that TDTMSB NPs have an excellent inhibitory effect on MDA‐MB‐231 malignant tumors (Figure 5d,e; Figure S16, Supporting Information). These results are mainly attributed to the large number of ROSs (^1^O_2_, OH, and O_2_^−^) and photothermal effects produced by TDTMSB NPs upon laser exposure. This type I/II PDT‐PTT synergistic treatment approach greatly improves the therapeutic effect of TDTMSB NPs on malignant tumors. Additionally, the body weight and major organs (heart, liver, spleen, lung, and kidney) of BALB/c nude mice in each group were recorded and collected to evaluate the biological safety of TDTMSB NPs. As shown in Figure 5f, there was no significant difference in the body weight of mice across groups. Additionally, hematoxylin and eosin (H&E) staining of major organs revealed no obvious damage to the heart, liver, spleen, lung, and kidney tissues after various treatments. The hemolysis test showed that A did not exhibit significant hemolysis. These results provide strong evidence that TDTMSB NPs possess good biological safety in vivo (Figure 5g). Overall, TDTMSB NPs demonstrate a highly synergistic effect in combined type I/II PDT and PTT for malignant tumors, while also demonstrating excellent biological safety in living organisms. Consequently, they hold significant promise as a phototheranostic agent.

## 3 Conclusion

In summary, we have dexterously designed and expediently synthesized a versatile amphipathic AIE phototheranostic system to implement a “3+2” cooperation pattern for synergistic phototheranostics in malignant tumors. This system encompasses NIR I/II fluorescence imaging, photoacoustic imaging, photothermal imaging, and image‐guided Type I/II PDT‐PTT synergistic phototherapy. The target compounds (TMSB, TTMSB, and TDTMSB) were synthesized through a Knoevenagel coupling reaction between 2‐methyl 1‐ (3‐sulfonatepropyl)‐benzothiazolium (MSB) and triphenylamine derivatives (4‐(N,N‐diphenylamino)benzaldehyde, 5‐(4‐(diphenylamino)phenyl)thiophene‐2‐carbaldehyde, and 5 (4‐(diphenylamino)phenyl)‐[2,2'‐bithiophene]‐5‐carbaldehyde). These molecules possessed an ingenious rotor twisted structure, D‐A conformation, and distinctive AIE characteristics. Thereinto, TDTMSB exhibited long emission wavelength, large Stokes shift (300 nm), and small ΔΕ_ST_ values compared to TMSB and TTMSB. Subsequently, TDTMSB was selected for further investigation and successfully formulated into TDTMSB NPs with good biocompatibility and stability via self‐assembly in DMSO/water mixed solution. Notably, such NPs exhibit remarkable ROSs generation capability (^1^O_2_, OH, and O_2_^−^), possess a photothermal conversion efficiency exceeding 40.5%, and specifically target the lysosomes of tumor cells. Furthermore, in vitro experiments demonstrated that TDTMSB NPs have no cytotoxicity up to a concentration of 80 µm, possess good fluorescence imaging performance, and exert an excellent inhibitory effect on tumor growth. Also, it can regulate the expression of pro‐apoptosis factors (caspase‐3 and bax) and inhibit apoptosis factor (bcl‐2) under light conditions. Moreover, in vivo studies demonstrated the capability of TDTMSB NPs to facilitate multimodal imaging, encompassing NIR I/II fluorescence imaging, photoacoustic imaging, and photothermal imaging. Notably, these NPs presented excellent efficacy in imaging‐guided Type I/II PDT‐PTT synergistic phototherapy, achieving high‐efficiency antitumor effects via ROSs generation and localized heating produced by laser irradiation. Consequently, TDTMSB emerges as a highly promising candidate for multimodal image‐guided phototherapeutic applications in cancer.

## Conflict of Interest

The authors declare no conflict of interest.

## Supporting information



Supporting Information

## Data Availability

The data that support the findings of this study are available from the corresponding author upon reasonable request.
