# Supplementary material for: A “3+2” Cooperation Pattern of Amphipathic AIE Phototheranostic System for Multimodal Image‐Guided Synergistic Type I/II Photodynamic‐Photothermal Therapy
Source: Adv Sci (Weinh). 2025 Sep 11;12(39):e07956. doi: 10.1002/advs.202507956 (PMC12533199; doi:10.1002/advs.202507956)
Supplement: Supplementary file 1 — Supporting Information [file ADVS-12-e07956-s001.doc]

*Supporting Information*

**A “3+2ˮ Cooperation Pattern of** **Amphipathic** **AIE Phototheranostic System for** **Multimodal Image-Guided Synergistic Type I/II Photodynamic-Photothermal Therapy**

*Haijun Ma, Yibo An, Yuanyuan Han, Feifan Zhao, Yunfei Zuo, Guokang He, Zhixiang Lu*,* *Ryan T. K. Kwok*, Jianwei Sun, Jacky W. Y. Lam, Yen Wei* and Ben Zhong Tang**

Prof. H. Ma., Ms. Y. Han., Ms. F. Zhao

Key Lab of Ministry of Education for Protection and Utilization of Special Biological Resources in Western China, School of Life Sciences, Ningxia University, Yinchuan 750021, China.

Dr. Y. An., Dr. Z. Lu

State Key Laboratory of Cellular Stress Biology & Fujian Provincial Key Laboratory of Innovative Drug Target Research, School of Pharmaceutical Sciences, Xiamen University Xiamen 361102, China.

E-mail: zhixiangl@xmu.edu.cn

Dr. Y. Zuo., Dr. G. He., Prof. R. T. K. Kwok., Prof. J. Sun, Prof. J. W. Y. Lam., Prof. B. Z. Tang

Department of Chemistry, Hong Kong Branch of Chinese National Engineering Research Center for Tissue Restoration and Reconstruction, Division of Life Science, State Key Laboratory of Molecular Neuroscience, and Department of Chemical and Biological Engineering, The Hong Kong University of Science and Technology, Clear Water Bay, Kowloon, Hong Kong 999077, China.

E-mail: tangbenz@cuhk.edu.cn; chryan@ust.hk

Prof. B. Z. Tang

School of Science and Engineering, Shenzhen Institute of Aggregate Science and Technology, The Chinese University of Hong Kong, Shenzhen (CUHK-Shenzhen), Guangdong, 518172, P.R. China.

Prof. Y. Wei

MOE Key Laboratory of Bioorganic Phosphorus Chemistry and Chemical Biology, Department of Chemistry, Tsinghua University, Beijing 100084, China.

E-mail: weiyen@mail.tsinghua.edu.cn

Keywords: Amphipathic aggregation-induced emission, Phototheranostic, Multimodal imaging, Type I/II photodynamic therapy, Photothermal therapy

**Contents**

1. Experimental Section

2. Additional experimental data

Figure S1. The preparation of MSB, TMSB, TTMSB and TDTMSB.

Figure S2. ^1^ H NMR spectrum of MSB.

Figure S3. ^1^ H NMR spectrum of TMSB.

Figure S4. ^1^ H NMR spectrum of TTMSB.

Figure S5. ^1^ H NMR spectrum of TDTMSB.

Figure S6. MALDI-TOF-MS spectrum of TMSB.

Figure S7. MALDI-TOF-MS spectrum of TTMSB.

Figure S8. MALDI-TOF-MS spectrum of TDTMSB.

Figure S9. The fluorescence lifetime of TMSB, TTMSB, and TDTMSB in the aggregate state.

Figure S10. The fluorescence lifetime of TDTMSB NPs.

Figure S11. The relative fluorescence intensity of TDTMSB NPs under continuous light activated for 1 h.

Figure S12. ROSs generation capacity of TDTMSB NPs inside tumor cells are determined by DCFH probe.

Figure S13. The ROSs generation rate of TDTMSB NPs are evaluated by a chemical process.

Figure S14. The survival rate and apoptosis rate of MDA-MB-231 cells incubated with TDTMSB NPs at various concentrations for 24 h via flow cytometry assay.

Figure S15. The temperature curve of tumor region in nude mice treated with PBS and TDTMSB NPs.

Figure S16. Photographs of MDA-MB-231 tumor-nude mice after treatments with saline, saline + Laser, TDTMSB NPs and TDTMSB NPs + Laser, respectively.

Figure S17. Evaluation of hemolysis in TDTMSB NPs.

**1.** **Experimental Section**

**1.1 Materials:** 5'-(4-(diphenylamino)phenyl)-[2,2'-bithiophene]-5-carbaldehyde 5-(4-(diphenylamino)phenyl)thiophene-2-carbaldehyde was purchased from Jilin Chinese Academy of Sciences-Yanshen Technology Co., Ltd. propanesultone and 2-methylbenzothiazole was obtained from Shanghai Aladdin Bio-Chem Technology Co.,Ltd. (Shanghai, China). Dichloromethane, trichloromethane, tetrahydrofuran, acetonitrile, ethanol and methanol, petrol ether, and ethyl acetate were purchased from Sinopharm Chemical Reagent Co., Ltd. (Beijing, China). Pyridine, N, N-dimethylformamide were got from J&K Scientific Ltd. (Beijing, China). Calcein AM, Annexin V-FITC and propidium iodide were obtained from Beijing Solarbio Science Technology Co.,Ltd. Hoechst 33258 and BODIPY 493/503 were purchased from Thermo Fisher Scientific.The mitotracker green and lysotracker green were gained from Beyotime Biotechnology. The hydroxyphenyl fluorescein (HPF) and dihydrorhodamine123 (DHR123) and 2',7'-dichlorodihydrofluorescein diacetate (DCFH-DA) were obtained from Maokang Biotechnology Co., Ltd (Shanghai, China). Dulbecco’s Modified Eagle’s Medium (DMEM) was acquired form Procell Life Science&Technology Co.,Ltd. Fetal bovine serum, penicillin, and streptomycin were obtained from Gibco. All experiment reagents were purchased from commercial suppliers without further purification.

**1.2 Measurements and Characterization:** ^1^H NMR and ^13^C NMR spectra were determined by a JEOL JNM-ECA400 (Tokyo, Japan) using DMSO-D6 as the solvent. Mass spectrometry was measured with a matrix-assisted laser desorption/ionization time-of-flight mass spectrometer (MALDI-TOF MS, Shimadzu, Japan). Fluorescence spectra were measured with a RF-5301 fluorescence spectrophotometer (Shimadzu, Japan). The UV−vis absorption spectra were recorded on a on a TU-1810PC (Beijing Purkinje General Instrument Co. Ltd, China). Transmission electron microscopy images were gained by a Hitachi 7650B (Tokyo, Japan) microscope operating at acceleration voltage of 80 kV. The hydrodynamic diameter and Zata potential were employed with a Zetasizer Nano ZSI (Malvern Instruments, Malvern, UK). Confocal images were collected by a Leica STELLARIS5 confocal laser scanning microscope (STELLARIS5, Germany). Cell viability was tested using a Multimode Plate Reader (PerkinElmer). Photoacoustic data was detected by a photoacoustic imaging system (Endra's Nexus 128 Fully 3-D Photoacoustic CT Scanner). NIR I/II fluorescence imaging were recorded by NIR II imaging system in vivo and IVIS Lumina S5.


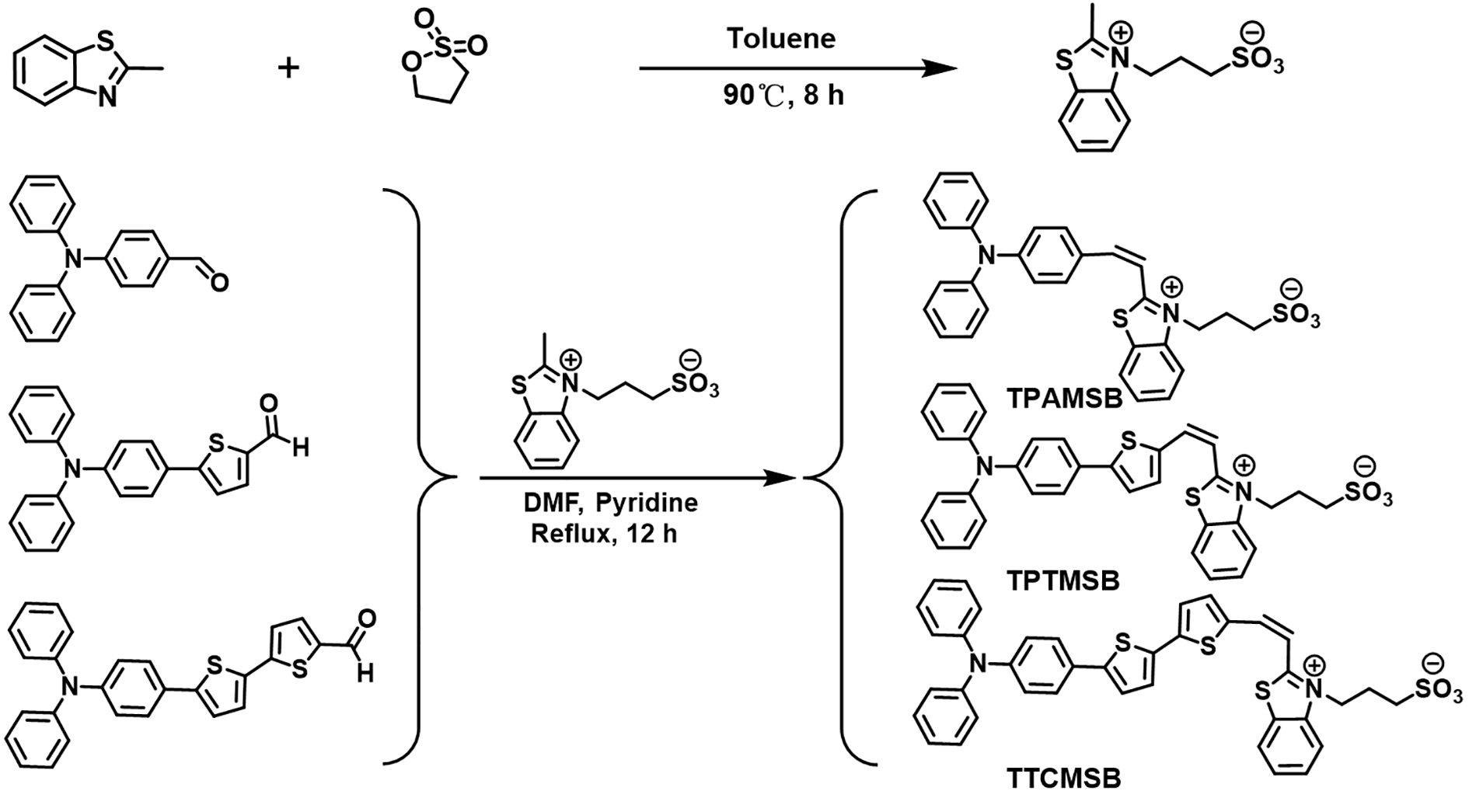


**Figure S1.** The preparation of MSB, TMSB, TTMSB and TDTMSB.

**1.3. Synthesis of** **TMSB, TTMSB and TDTMSB:** The 2-methylbenzothiazole (745 mg, 5 mmol) and propanesultone (610 mg, 5 mmol) were dissolved in toluene and heated at 90℃ for 12 h under N_2_ to prepare the intermediate product of 2-methyl-1-(3-sulfonatepropyl)-benzothiazolium (MSB). ^1^H NMR (400 MHz, DMSO-D6) δ 7.48-7.40 (m, 2H), 7.24-7.18 (m, 1H), 7.12 (t, *J* = 6.2 Hz, 1H), 4.83 - 4.80 (m, 2H), 3.46 (s, 3H), 3.41 (t, *J* = 5.6 Hz, 2H), 2.81 (m, 2H). Then the 4-(N,N-diphenylamino)benzaldehyde (526 mg, 2 mmol), 5-(4-(diphenylamino)phenyl)thiophene-2-carbaldehyde (710 mg, 2 mmol). 5'-(4-(diphenylamino)phenyl)-[2,2'-bithiophene]-5-carbaldehyde (875 mg, 2 mmol), 2-methyl-1-(3-sulfonatepropyl)-benzothiazolium (570 mg, 2 mmol) were respectively dissolved in dimethyl formamide solution (50 mL), and pyridine (0.2 mL) was used as a catalyst. Then the reaction was heated at 150 ℃ in an N_2_ atmosphere for 12 h, and extracted with CHCl_2_. Whereafter, the sample was refined by column chromatography on silica gel with dichloromethane/methyl alcohol (v/v = 30/1), and TMSB, TTMSB and TDTMSB were successfully gained (Figure S1).

**1.4 Synthesis of** **TDTMSB NPs:** As-prepared TDTMSB (6.1 mg) is dissolved in dimethyl sulfoxide (DMSO, 2 mL) and treated by an ultrasonic apparatus for 5 min. The ultrapure water (8 mL) was added and sonicated for 30 min, then the mixture solution was addressed through dialysis tube (1000 Da) to get the target sample TDTMSB NPs.

**1.5 Measurements of Reactive Oxygen Species (ROSs):** X-band electron paramagnetic resonance (EPR) technology was able to effectively estimate ROSs production. Specifically, 2,2,6,6-tetramethylpiperidine (TEMP) acted as a capture agent specifically for singlet oxygen and formed TEMPO when combined with ^1^O_2_ produced by TDTMSB under illuminate conditions, the dark as the control group. The formed TEMPO was able to generate a paramagnetic signal and thus can be detected by EPR. Also the EPR signal intensity usually indicates ^1^O_2_ generation. Simultaneously, 5,5-dimethyl-1-pyrroline N-oxide (DMPO) and 5-tert-butoxycarbonyl-5-methyl-1-pyrroline N-oxide (BMPO) are used as free radical catching agent for the hydroxyl radical (·OH) and superoxide radical (·O_2_^−^). In addition, the ^1^O_2_ production was quantitatively detected by a chemical approach, which mainly used rose bengal (RB) as the standard reagent and 9,10-anthracenediyl-bis(methylene)-dimalonic acid (ABDA) as the binding reagent, and then determined the attenuation degree of ABDA under light conditions for different times to calculate ROSs production. Specifically speaking, 250 μL of ABDA solution was separately added into 250 μL of TDTMSB NPs and RB solution, and treated by a white light (40 mW/cm^-2^) for different time. Then the absorption value of ABDA at 378 nm were recorded by TU-1810PC, and the ROSs production efficiency was calculated according to the equation {Φ_NPs_ = Φ_RB_ × K_NPs_ × A_RB_ / (K_RB_ × A_NPs_)}. Φ_NPs_ and Φ_RB_ are the ^1^O_2_ generation of TDTMSB NPs and RB, K_NPs_ and K_RB_ are the degradation constant TDTMSB NPs and RB, A_NPs_ and A_RB_ are the absorption value of TDTMSB NPs and RB, respectively. Furthermore, the hydroxyl radical (·OH) and superoxide radical (·O_2_^−^) generated by TDTMSB NPs are quantitatively measured through hydroxyphenyl fluorescein (HPF) and dihydrorhodamine123 (DHR123). Moreover, DCFH assays is performed to further prove the total ROSs generation of TDTMSB NPs and Briefly, the fluorescence intensity changes of DCFH at 580nm under different treatment conditions are recorded by fluorescence spectrometer. DCFH assays are also vigorously confirmed whether such NPs can able to produce ROSs inside the cells. In detail, the cells treated with different concentration of TDTMSB were illuminated by a white light, untreated cells and without irradiation as the control group. Then the cells were incubated with DCFH-DA for 30 min and washed by PBS for 2-3 time. Next the fluorescence images were collected by confocal laser scanning microscopy (CLSM, Leica STELLARIS5).

**1.6 Photothermal Effect of TDTMSB NPs:** The TDTMSB NPs (20 uM, 300 μL) and PBS (300 μL) are collected in 1.5 mL centrifuge tube and irradiated by a 630 nm laser (300 mW cm^-2^) for different times (0-300 s). Then the temperature variation of TDTMSB NPs and PBS at each observing time are recorded by a Fotric343 infrared thermal imager. In addition, the temperature changes of TDTMSB NPs are detected during continuous irradiation with and without laser for 300 s and five cycle periods are recorded at each interval of 600s as a cycle period, which to further investigate its photothermal stability.

**1.7 Cytotoxicity of TDTMSB NPs:** The MDA-MB-231 and MCF-7 were cultured in DMEM containing fetal bovine serum (10%) and penicillin/streptomycin (1%) at 37 °C in a humidified atmosphere with 5% CO_2_. Then they were planted in 96-well plates (5×10^3^ cells/well) and cultured for overnight. And replaced with fresh DMEM containing the different concentrations of TDTMSB NPs (1.25, 2.5, 5, 10, 20 and 40 μM) incubated cells for 24 h. Whereafter, colourless DMEM containing CCK-8 (10%) was added and treated for 1-2 h. The cell viability was collected by a microplate reader. Beside, the cells were treated using the same methods as described above, then cell survival rate and apoptosis rate were measured by flow cytometry.

**1.8 Confocal Images of TDTMSB:** MDA-MB-231 cells with a density of 1×10^5^/well were seeded in confocal dish, after 24 h. The culture medium was substituted with fresh DMEM containing 10 μM of TDTMSB, which co-incubated cells for various time (2, 4, 8 and 12 h). When reaching the set time, the DMEM was removed and the PBS solution was used to wash the cells. Eventually, cloorless DMEM was added in confocal dish with cells and fluorescence images was collected by CLSM. The excitation wavelength was 525 nm, the emission wavelength was 825 nm. In addition, cells were treated with of TDTMSB (10 μM) for 4 h, then hoechst 33258 (50 μg mL^−1^ for 20 min), lyso-tracker green (50 μg mL^−1^ for 30 min), mito-tracker green (50 μg mL^−1^ for 20 min) and BODIPY 493/503 (100 μg mL^−1^ for 25 min) were applied to label nucleus, lysosome, mitochondria and lipid droplet, respectively. Also, cells were washed with PBS buffer solution for 3 time and confocal photographs were obtained through a Leica STELLARIS5. The regions of interest (ROIs) were analyzed and calculated by ImageJ Software. Furthermore, cellular metabolism of TDTMSB was investigated by confocal technology. Simply, MDA-MB-231 cells incubated with TDTMSB were cultured and separated by successive generations. Then different progeny cells were fixed by cell fixative solution and imaged using a CLSM.

**1.9** **Evaluating phototherapy effect in *vitro*:** The CCK-8 assays were selected to evaluate phototherapy effect. Briefly, MDA-MB-231 cells were cultured in 96-well plates and handled with TDTMSB (1.25, 2.5, 5, 10, 20 and 40 μM). After 6 h, they were illuminated though a white light (40 mW cm^-2^) and incubated with fresh culture medium. Then cells vibility was recorded by a microplate reader through CCK-8 kit. Without treated group as a control group. Simultaneously, AM/PI stained with live/dead cells assays was used to further verify phototherapy effect of TDTMSB. MDA-MB-231 cells were seeded in confocal dish and treated with the various concentration of TDTMSB (5 and 10 μM) as well as irradiated by a white light (40 mW cm^-2^) for 10 min. Then the cells were incubated with fresh DMEM for 12-24 h, and followed by that they were handled with colourless DMEM containing AM/PI staining solution for 20 min. after that, the cells were rinsed with PBS and imaged via a CLSM. The excitation wavelength of AM and PI were 488 nm and 534 nm, that emission wavelength were 525 nm and 630 nm, respectively. Besides, we used flow cytometry technique to further explore phototherapy effect of TDTMSB. Based on the above treatment method, the cells were successively treated with Annexin V-FITC and PI for 5 min. After centrifugation and washing, the cells were resuspended and measured by a flow cytometry. The excitation wavelength of Annexin V-FITC and PI were 488 nm and 525 nm, respectively.

**1.10 Animal Experiments****:** BALB/C mice aged 4–6 weeks are purchased from GemPharmatech Co.,Ltd. (Jiangsu, China). All animal experiments are implemented in accordance with related provision of the laboratory animal center of Ningxia University (Animal Welfare Assurance no. NXU-2023-041, Ningxia, China). Tumor mice model is constructed through subcutaneous injection with 100 μL of MDA-MB-231 cells (2.5 × 10^6^).

**1.11 Fluorescence Imaging, Photoacoustic Imaging and** **Photothermal Imaging *In Vivo*:** The grafted MDA-MB-231 tumor cells of BALB/C-nu mice were disposed with 100 μL of TDTMSB NPs sample solution (20 μM) through an intratumor injection method for different time interval (0.5, 2, 4, 8, 12, 24, 36 and 48 h). The tumor BALB/C-nude mice were anesthetized by isoflurane (2%), then pictured by a IVIS spectrum in vivo imaging system (The excitation wavelength: 560 nm, Emission wavelength: 700-890 nm) and Photoacoustic Scanner system. Furthermore, the tumor mice were treated with TDTMSB NPs (100 μL, 20 μM) for 2 h and imaged by a Fotric343 infrared thermal imager to obtain photothermal imaging. In addition, to further explore the organism distributions of TDTMSB NPs. The mice were anatomized after injected TDTMSB NPs for 24 h, and the major organs (heart, liver, spleen, lung and kidney and tumor) were collected and rinsed by PBS buffer solution, then imaged by a IVIS spectrum system.

**1.12 PDT-PTT effect of TDTMSB NPs in Vivo:** The xenografted MDA-MB-231 tumor cells of BALB/C nude mice were randomly divided into four groups, its named Saline, Saline + laser, TDTMSB NPs, and TDTMSB NPs + laser group and each group have 6 mice. The mice in Saline and Saline + laser group were treated with Saline (100 μL) by intratumoral injection, and other mice in two groups were disposed with TDTMSB NPs. Then the mice in Saline + laser and TDTMSB NPs + laser group were irradiated with 630 nm Laser (300 mW cm^-2^) for 10 min, and the treatment was given once every other day for a total of 7 times (15 days). The tumor volume and bodyweight of the mice was determined before each treatment. **1.13 H&E assays:** The MDA-MB-231 tumor mice were treated for 15 days and killed by a carbon dioxide inhalation method. Then the major organs (heart, liver, spleen, lung, and kidney) in mice were collected and fixed through 4% formalin solution for overnight. The fixed major organs were packed using paraffin and made into test samples with a thickness of 5 μm. Whereafter, the test samples were dyed with hematoxylin and eosin and measured using an optical microscopy.

**Statistics:** The data are demonstrated as the mean ± standard deviation (S.D.). The *p < 0.05, **p< 0.01, and ***p< 0.001 is regard as statistically significant difference.

**2. Additional experimental data**


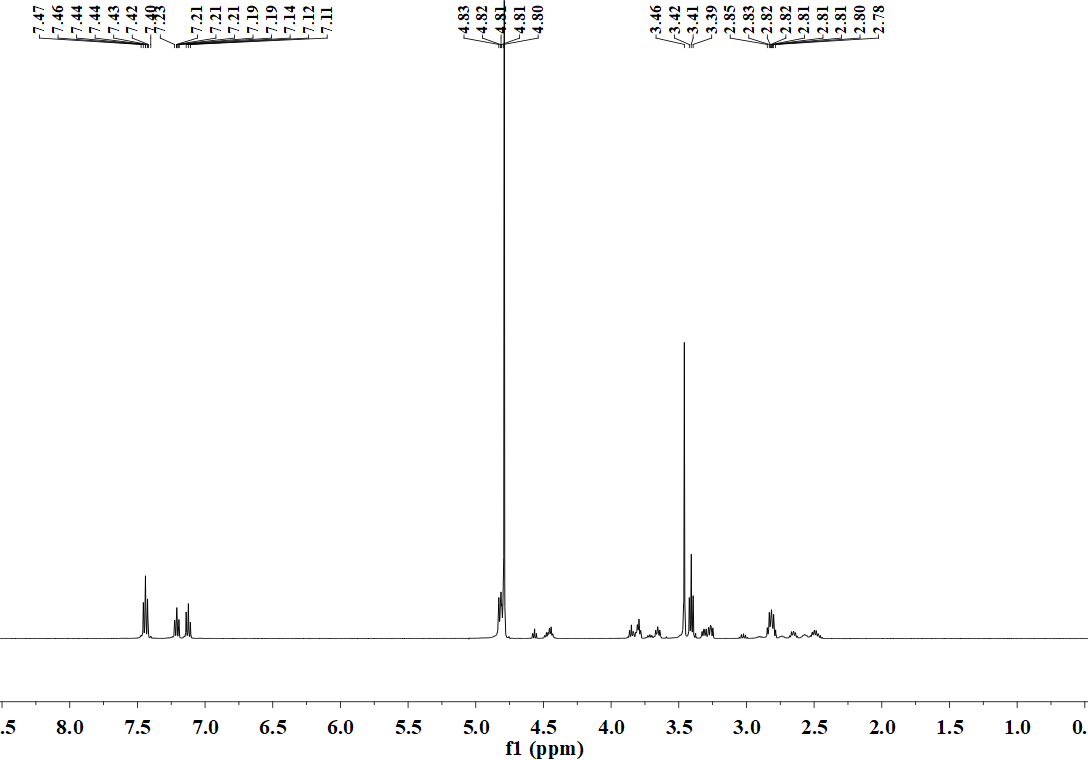


**Figure S2** The ^1^ H NMR spectrum of MSB

The ^1^H NMR spectrum of TDTMSB is measured by nuclear magnetic resonance spectrometer (JEOL JNM-ECA400) (Figure S1). ^1^H NMR (400 MHz, DMSO-d6) δ 7.48-7.40 (m, 2H), 7.24-7.18 (m, 1H), 7.12 (t, *J* = 6.2 Hz, 1H), 4.83 - 4.80 (m, 2H), 3.46 (s, 3H), 3.41 (t, *J* = 5.6 Hz, 2H), 2.81 (m, 2H).


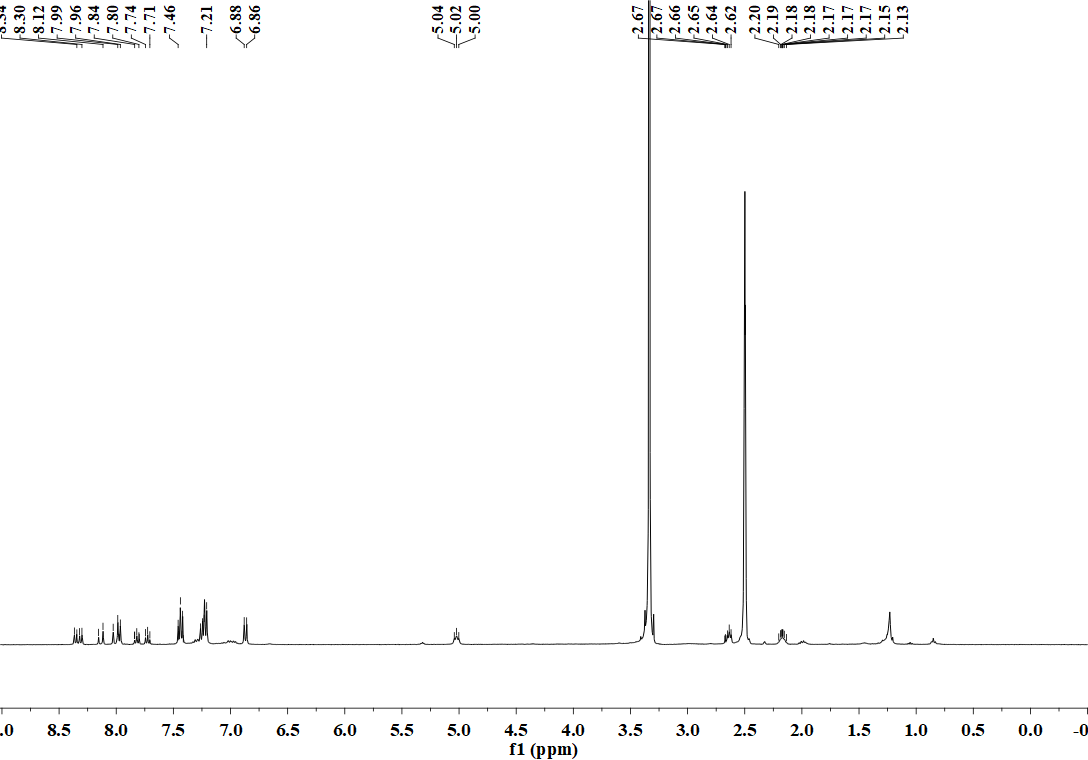


**Figure S3**. The ^1^ H NMR spectrum of TMSB.

^1^H NMR (400 MHz, DMSO-d6) δ 8.36 (d, *J* = 8.6 Hz, 1H), 8.31 (d, *J* = 8.5 Hz, 1H), 8.17 – 7.93 (m, 4H), 7.85 – 7.79 (m, 1H), 7.73 (t, *J* = 7.7 Hz, 1H), 7.48 – 7.40 (m, 4H), 7.27 – 7.19 (m, 6H), 6.87 (d, *J* = 8.7 Hz, 2H), 5.02 (t, *J* = 7.9 Hz, 2H), 2.68 – 2.61 (m, 2H), 2.23 – 2.11 (m, 2H).


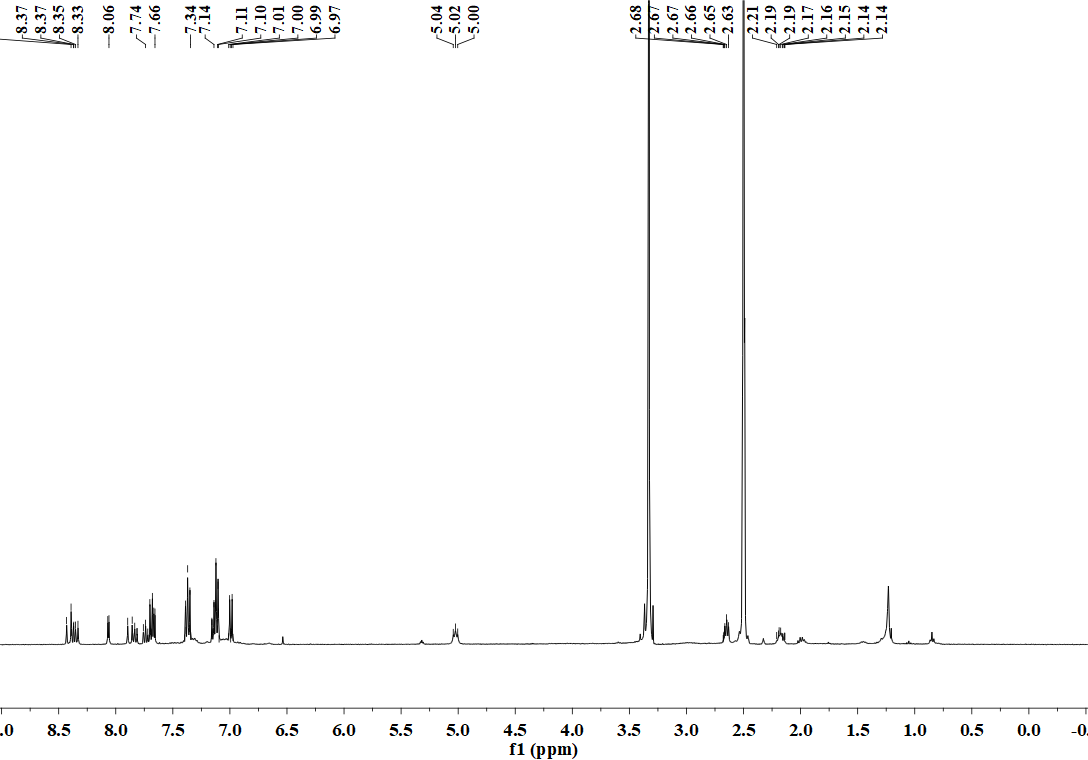


**Figure S4**. The ^1^ H NMR spectrum of TTMSB.

^1^H NMR (400 MHz, DMSO-d6) δ 8.44 – 8.32 (m, 3H), 8.06 (d, *J* = 4.1 Hz, 1H), 7.92 – 7.80 (m, 2H), 7.74 (t, *J* = 7.7 Hz, 1H), 7.71 – 7.65 (m, 3H), 7.40 – 7.34 (m, 4H), 7.17 – 7.09 (m, 6H), 7.02 – 6.97 (m, 2H), 5.02 (t, *J* = 7.9 Hz, 2H), 2.68 – 2.62 (m, 2H), 2.24 – 2.12 (m, 2H).


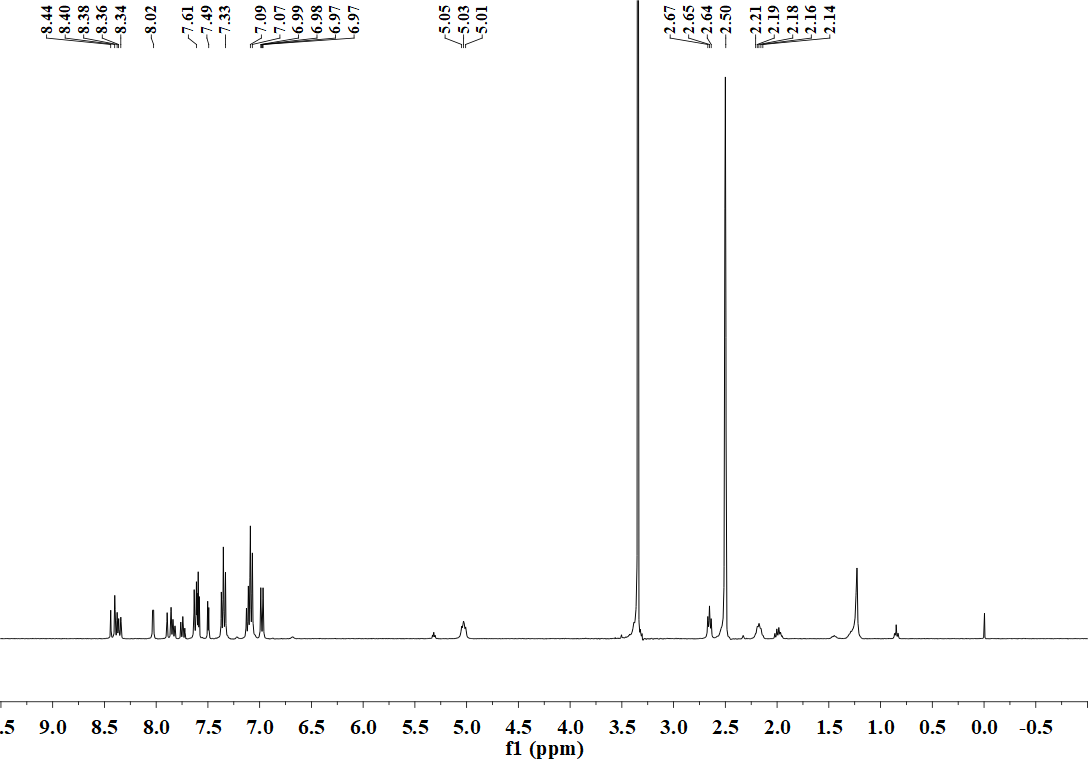


**Figure S5**. The ^1^ H NMR spectrum of TDTMSB.

^1^H NMR (400 MHz, DMSO-D6) δ 8.46-8.31 (m, 3H), 8.03 (d, *J* = 4.1 Hz, 1H), 7.91-7.80 (m, 2H), 7.74 (t, *J* = 7.7 Hz, 1H), 7.65-7.57 (m, 4H), 7.50 (d, *J* = 4.0 Hz, 1H), 7.35 (t, *J* = 7.9 Hz, 4H), 7.09 (m, 6H), 7.00-6.95 (m, 2H), 5.03 (t, *J* = 7.8 Hz, 2H), 2.65 (t, *J* = 6.6 Hz, 2H), 2.18 (m, 2H).


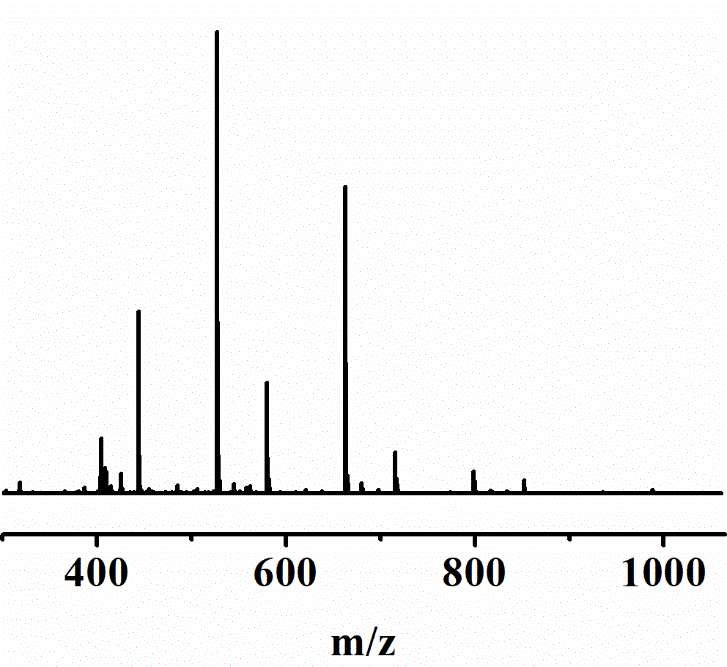


**Figure S6.** The mass spectrum of TMSB

In Figure S6, the mass spectrum of TMSB was collected by the Matrix-Assisted Laser Desorption/Ionization Time of Flight Mass Spectrometry (MALDI-TOF-MS). MS (M+H) [m/z]: 527.1435 (Calcd: 526.14). Anal. Calcd. for C_30_H_26_N_2_O_3_S_2_.

**Figure S7.** The mass spectrum of TTMSB

In Figure S7, the mass spectrum of TTMSB was detected by the MALDI-TOF-MS. MS (M+H) [m/z]: 609.6311 (Calcd: 609.13). Anal. Calcd. for C_34_H_28_N_2_O_3_S_3_.


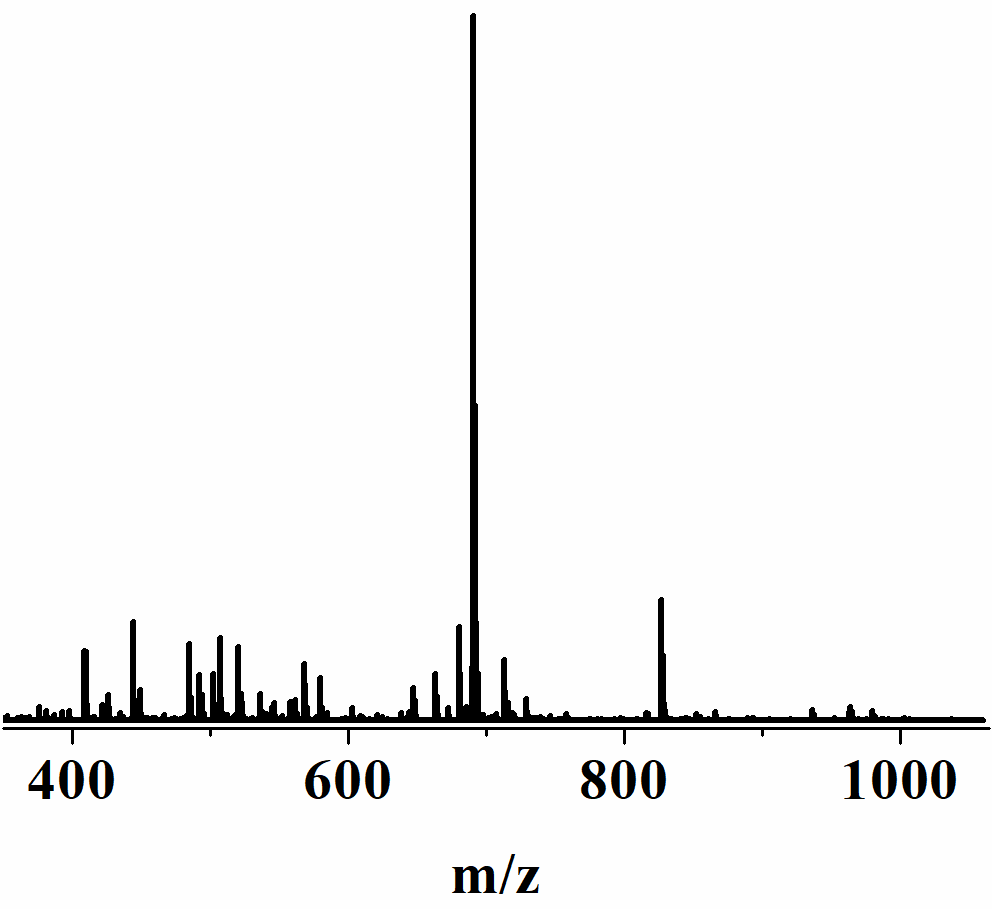


**Figure S8.** The mass spectrum of TDTMSB

In Figure S8, the mass spectrum of TDTMSB is recorded by MALDI-TOF-MS. MS (M+H) [m/z]: 691.1173 (Calcd: 691.11). Anal. Calcd. for C_38_H_30_N_2_O_3_S_4_.


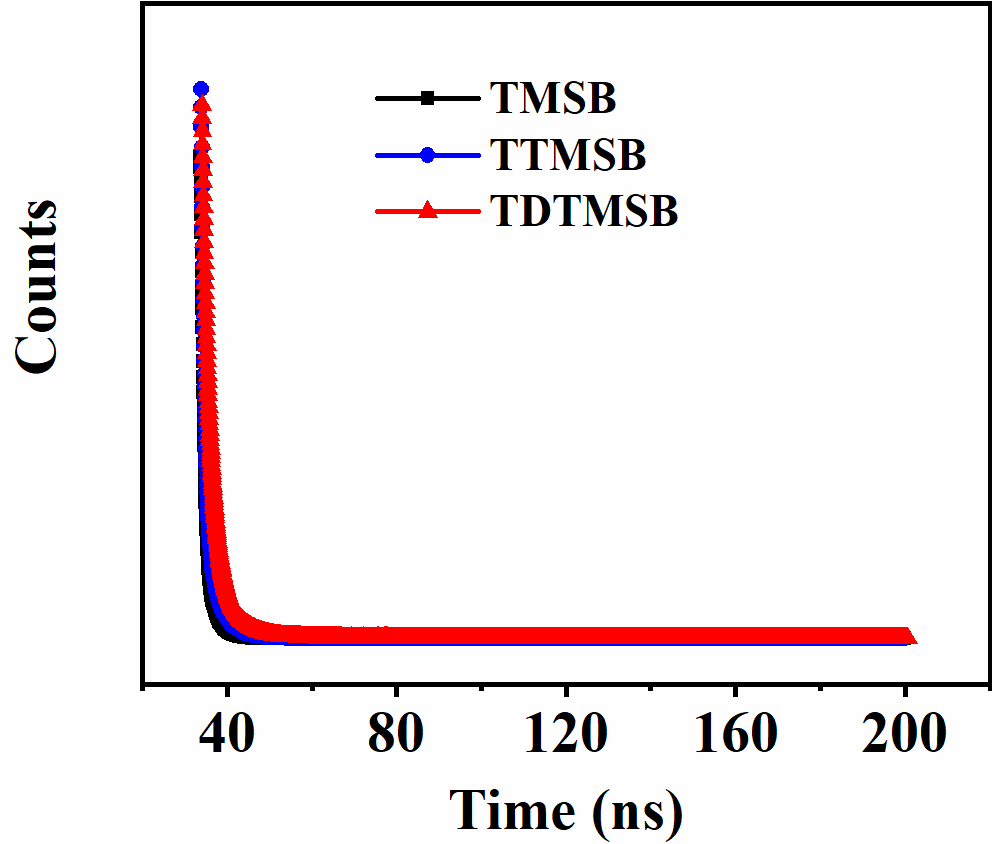


**Figure S9.** The fluorescence lifetime of TMSB, TTMSB, and TDTMSB in the aggregate state.


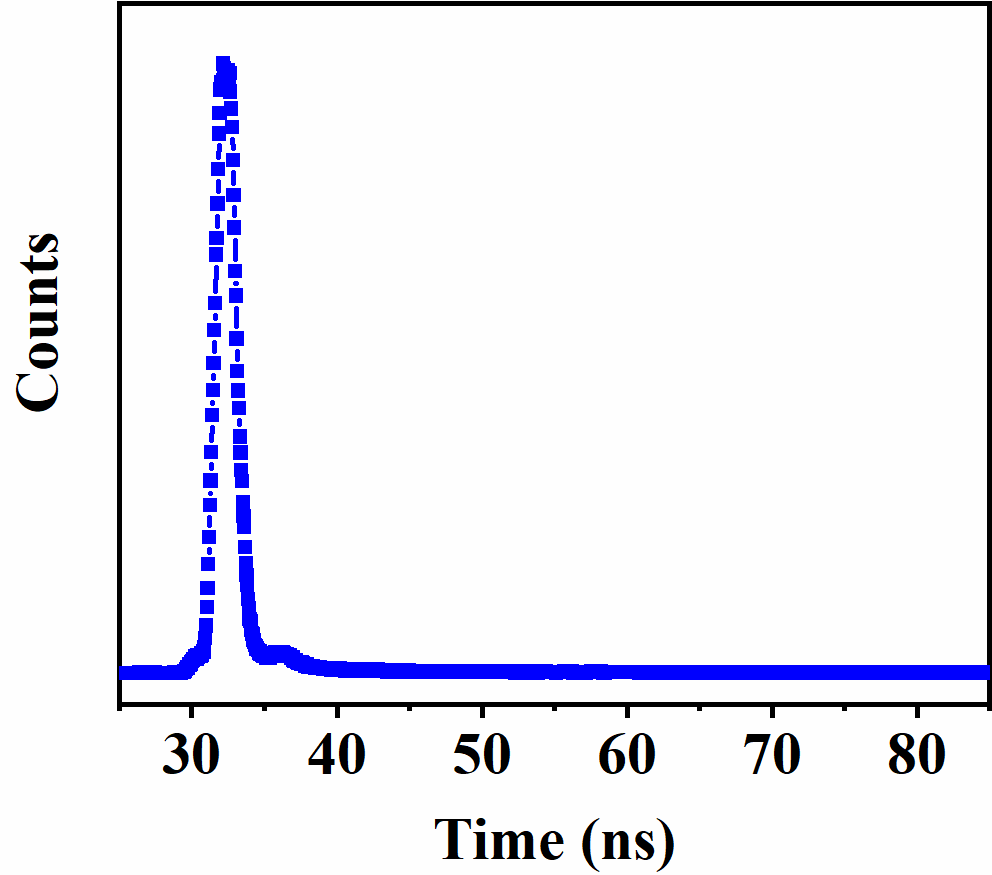


**Figure S10.** The fluorescence lifetime of TDTMSB NPs.


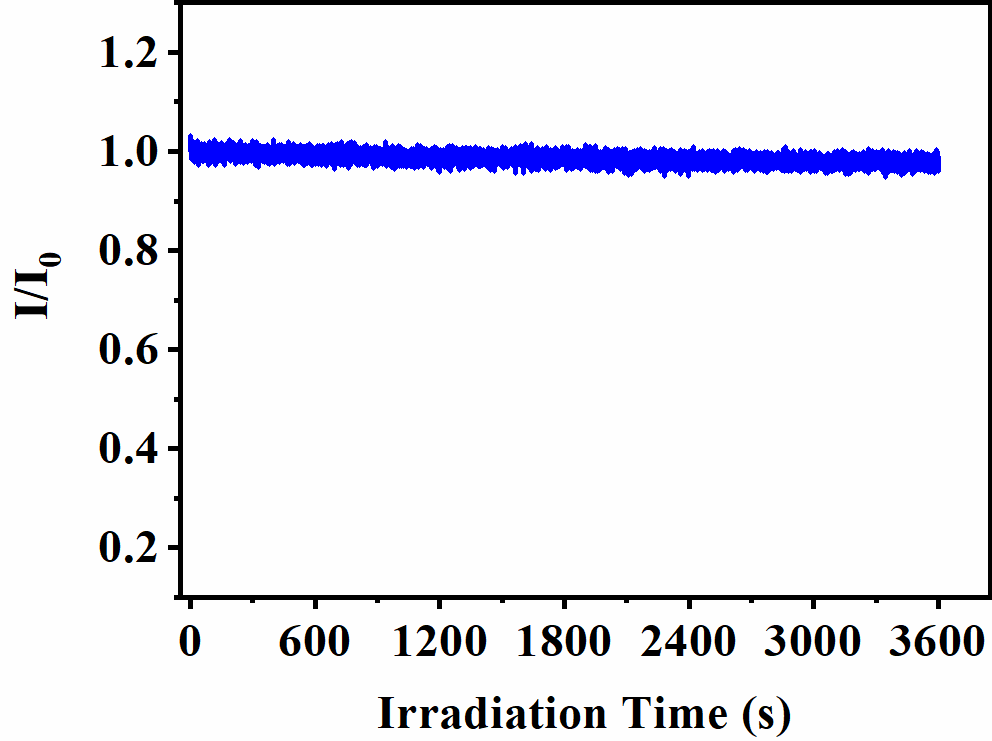


**Figure S11.** The relative fluorescence intensity of TDTMSB NPs under continuous light activated for 1 h.


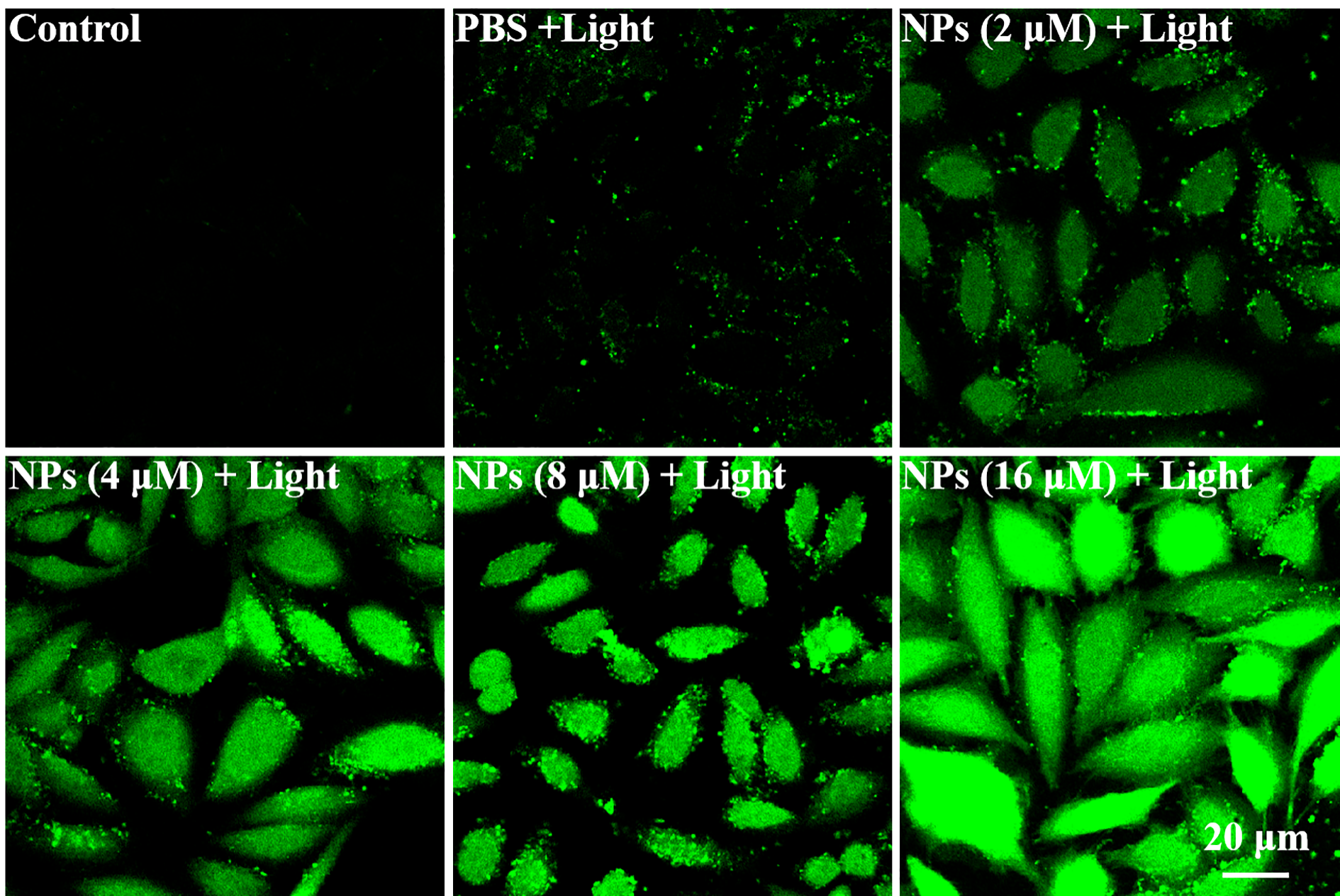


**Figure S12.** ROSs generation capacity of TDTMSB NPs inside tumor cells are determined by DCFH probe.


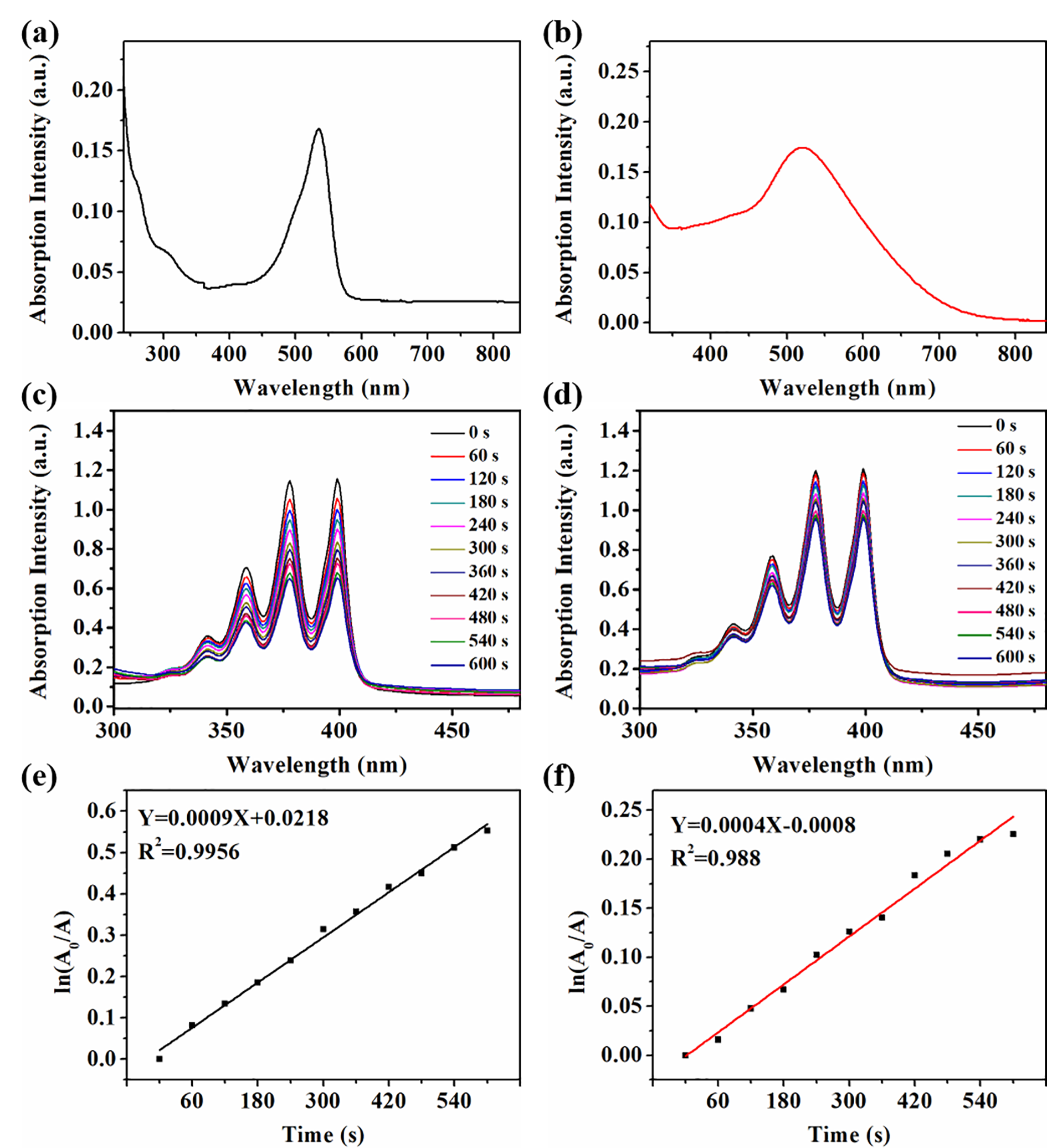


**Figure S13.** The UV absorption spectrum of (a) RB and (b) TDTMSB NPs. The photodegradation of ABDA in (c) rose-bengal (RB) and (d) TDTMSB NPs aqueous solution under light irradiation for different time (0-600 s). The photodegradation rate constant of the ABDA (380 nm) in aqueous solution of (e) RB and (f) TTCBTA NPs.


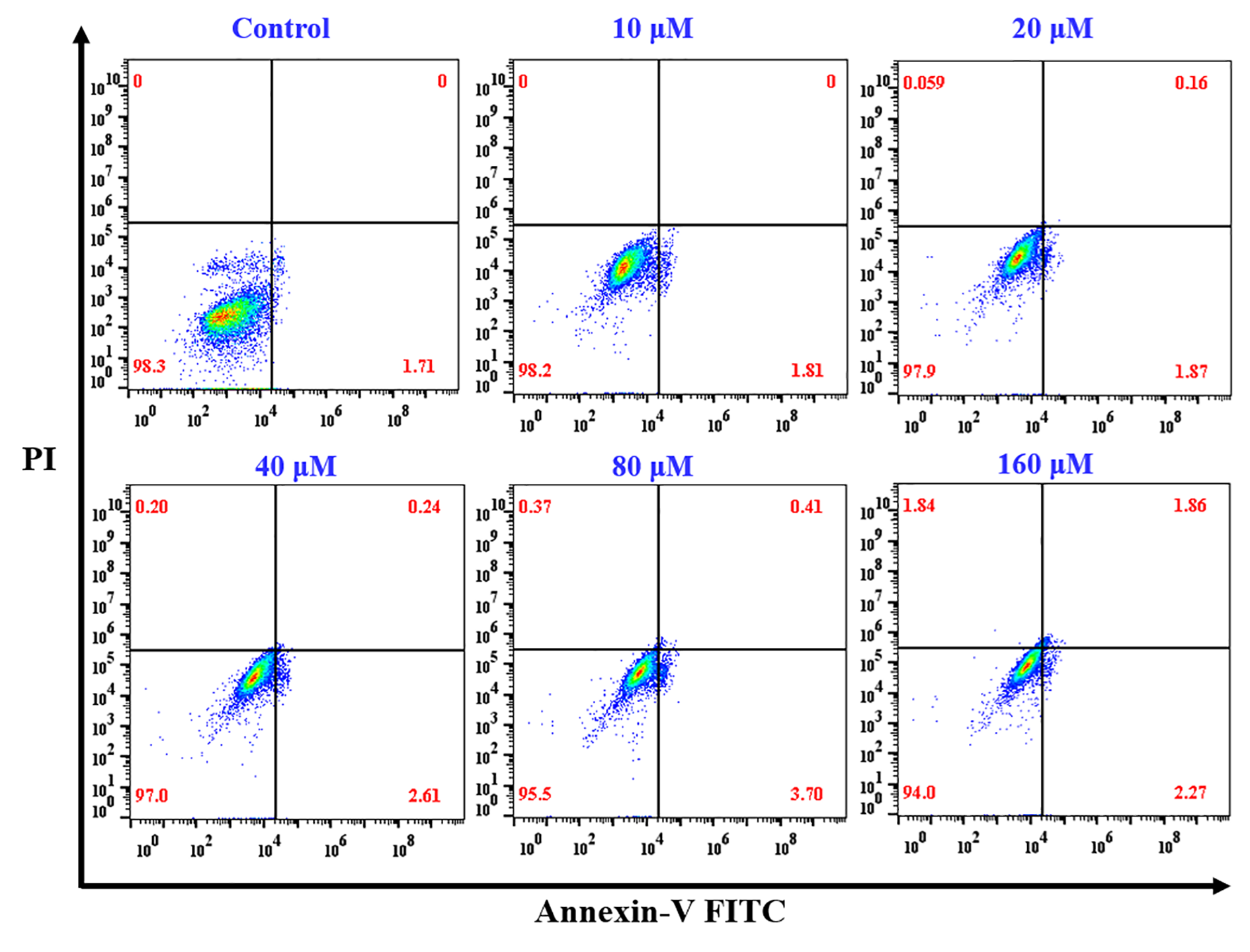


**Figure S14.** The survival rate and apoptosis rate of MDA-MB-231 cells incubated with TDTMSB NPs at various concentrations for 24 h via flow cytometry assay.


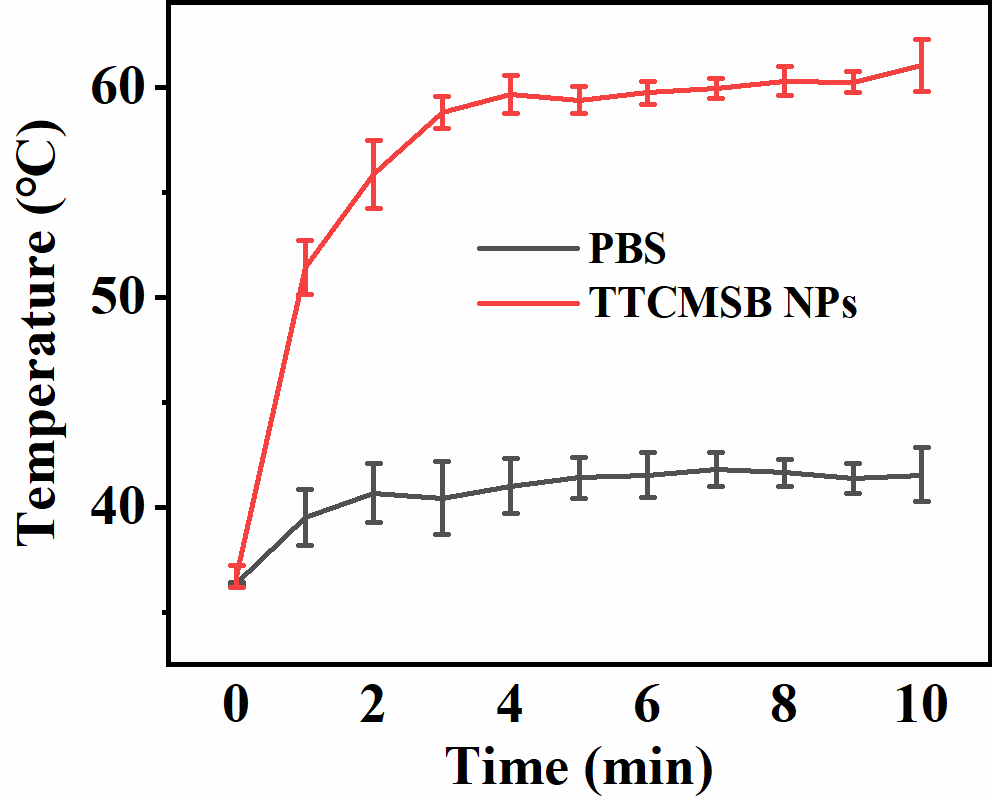


**Figure S15.** The temperature curve of tumor region in nude mice treated with PBS and TDTMSB NPs.


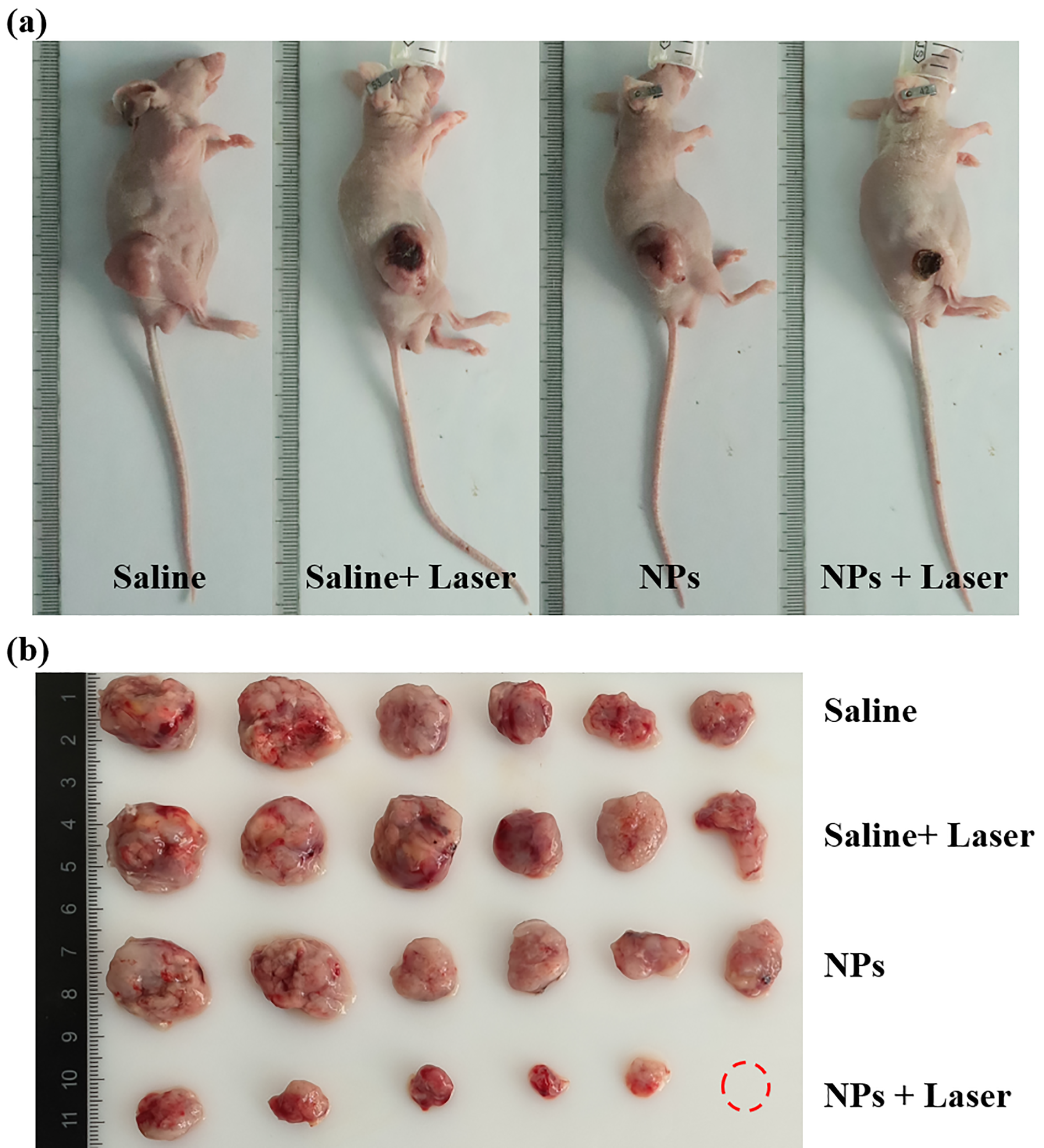


**Figure S16.** (a) Photographs of MDA-MB-231 tumor-nude mice and (b) the pictures of tumor in different group (saline, saline + Laser, TDTMSB NPs and TDTMSB NPs + Laser groups).


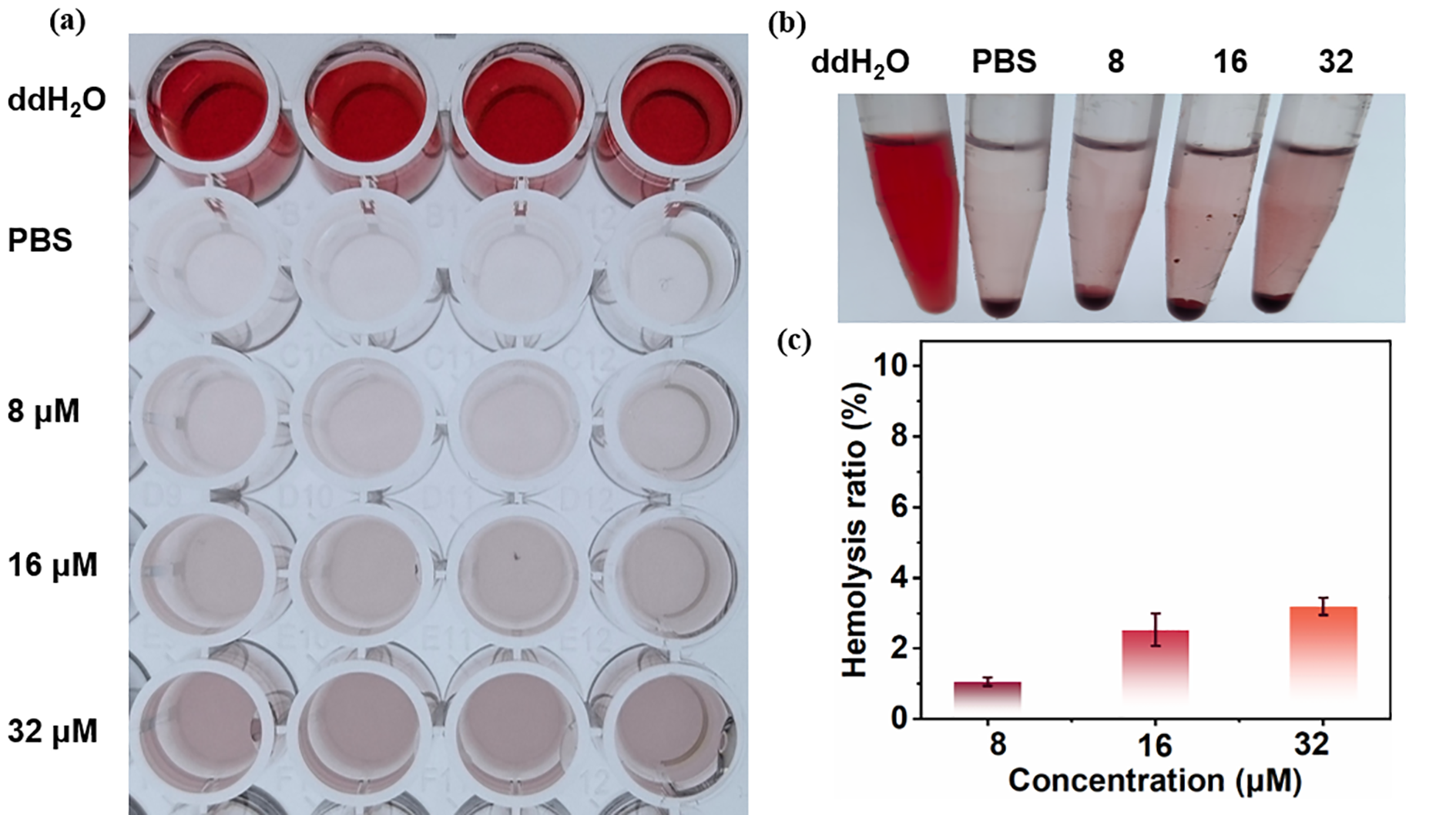


**Figure S17.** Evaluation of hemolysis in TDTMSB NPs. Assess the hemolytic activity of TDTMSB NPs using both (a) 96-well microtiter plates and (b) centrifuge tubes. (c) The quantitative analysis of the hemolysis ratio for TDTMSB NPs.
